# Porphyrin Dyes for Nonlinear Optical Imaging of Live Cells

**DOI:** 10.1016/j.isci.2018.05.015

**Published:** 2018-05-26

**Authors:** Anjul Khadria, Jan Fleischhauer, Igor Boczarow, James D. Wilkinson, Michael M. Kohl, Harry L. Anderson

**Affiliations:** 1 Department of Chemistry, Chemistry Research Laboratory, University of Oxford, Oxford OX1 3TA, UK; 2 Department of Physiology, Anatomy and Genetics, University of Oxford, Oxford OX1 3PT, UK

**Keywords:** Chemistry, Organic Synthesis, Imaging Methods in Chemistry, Nonlinear Optics

## Abstract

Second harmonic generation (SHG)-based probes are useful for nonlinear optical imaging of biological structures, such as the plasma membrane. Several amphiphilic porphyrin-based dyes with high SHG coefficients have been synthesized with different hydrophilic head groups, and their cellular targeting has been studied. The probes with cationic head groups localize better at the plasma membrane than the neutral probes with zwitterionic or non-charged ethylene glycol-based head groups. Porphyrin dyes with only dications as hydrophilic head groups localize inside HEK293T cells to give SHG, whereas tricationic dyes localize robustly at the plasma membrane of cells, including neurons, *in vitro* and *ex vivo*. The copper(II) complex of the tricationic dye with negligible fluorescence quantum yield works as an SHG-only dye. The free-base tricationic dye has been demonstrated for two-photon fluorescence and SHG-based multimodal imaging. This study demonstrates the importance of a balance between the hydrophobicity and hydrophilicity of amphiphilic dyes for effective plasma membrane localization.

## Highlights

• Amphiphilic porphyrin dyes with different hydrophilic head groups are synthesized
• Necessity of balance between hydrophobicity and hydrophilicity for membrane dyes
• Far-red to NIR absorbing dyes for multimodal and SHG-only imaging are presented
• SHG from a dye labeling the intracellular organelles of live cells is shown

## Graphical Abstract

**Figure:**
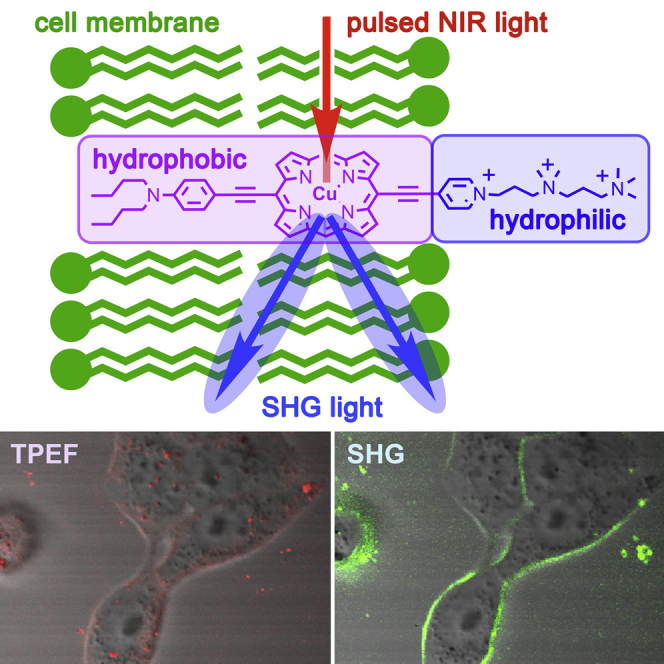

## Introduction

**Figure 1:**
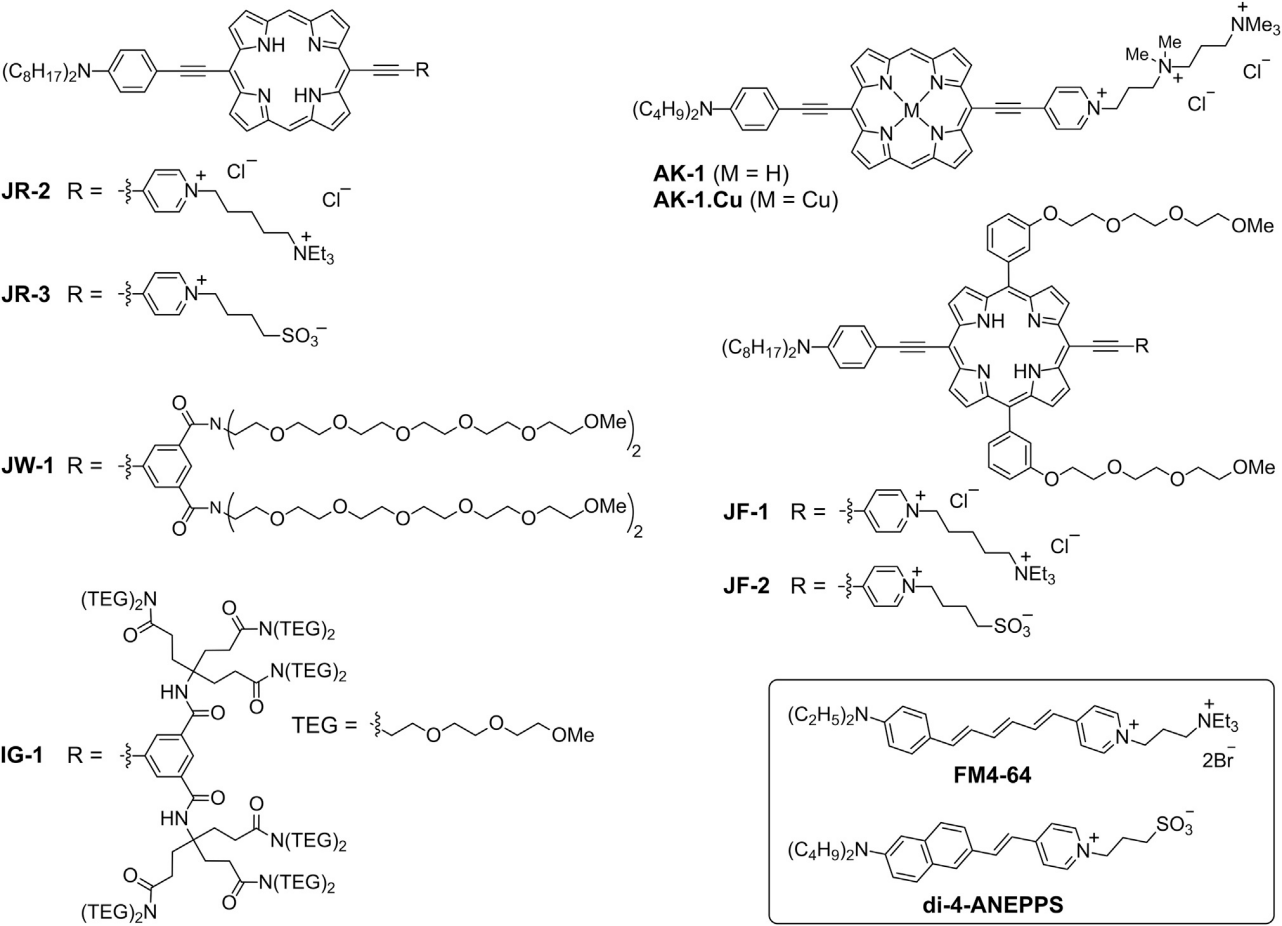
Chemical Structures of Dyes with Different Hydrophilic Head Groups

Nonlinear optical microscopies based on two-photon excited fluorescence (TPEF) and second harmonic generation (SHG) offer various advantages over linear optical microscopy, such as deep light penetration, less photodamage, and reduced background signal (Campagnola and Dong, 2011, Denk and Svoboda, 1997, Helmchen and Denk, 2006, Khadria et al., 2017, Pantazis et al., 2010, Pawlicki et al., 2009, Rau and Kajzar, 2008). Both TPEF and SHG have been established as robust tools for biological imaging, as well as for measuring membrane potentials of neurons *in vitro* and *ex vivo* (Benoren et al., 1996, Campagnola et al., 1999, Campagnola and Loew, 2003, Dombeck et al., 2005, Dombeck et al., 2004, Helmchen and Denk, 2006, Jiang et al., 2007, Kuhn et al., 2008, Nuriya et al., 2016, Nuriya et al., 2005, Zoumi et al., 2002). TPEF can be generated from a chromophore in homogeneous or non-homogeneous media alike, whereas SHG is generated only from non-centrosymmetric ensembles of chromophores, which makes it selective for dyes at interfaces. This selectivity is useful for imaging biological structures, such as plasma membranes (Campagnola et al., 1999, Campagnola and Dong, 2011, Doughty et al., 2013, Freund et al., 1986, Salafsky, 2007, Zoumi et al., 2002). SHG is also useful for measuring the membrane potential of excitable cells (Dombeck et al., 2005, Dombeck et al., 2004, Jiang et al., 2007, Jiang and Yuste, 2008, Millard et al., 2003). For membrane imaging, SHG has two major advantages over TPEF: (1) it does not require population of real excited states, and hence it can avoid the production of reactive oxygenated species or photochemistry and (b) no signals are given from isotropic media because SHG is generated only at interfaces (Reeve et al., 2010, Verbiest et al., 1997). Despite its advantages, SHG is not yet widely used for biological studies, whereas TPEF is exploited through many fluorescent dyes (Collins et al., 2008, Drobizhev et al., 2011, Ferrand et al., 2014, Helmchen and Denk, 2006, Nikolenko et al., 2007, Palmer et al., 2014, Pawlicki et al., 2009, Stosiek et al., 2003, Svoboda and Yasuda, 2006, Yuste and Denk, 1995). One of the major reasons why SHG is underutilized is the lack of suitable chromophores. Although TPEF and SHG are independent techniques, both require simultaneous use of two photons of equal energy, typically from a pulsed laser, and SHG and TPEF are often detected simultaneously. Until now, only one dye that gives SHG signals but no TPEF (Nuriya et al., 2016) has been reported. SHG signals tend to be weak, and not many dyes have been developed that possess high SHG efficiency, as characterized by the first-order hyperpolarizability, *β*_zzz_. The azo dye reported by Nuriya et al. gives similar or lower SHG signals than the styryl dye, **FM4-64** (*β*_zzz_ ≈ 1,100 × 10^−30^ esu at 800 nm in CHCl_3_) (Khadria et al., 2017), and exhibits lower voltage sensitivity (<5% per 100 mV) (Nuriya et al., 2016). We have previously demonstrated that highly electronically conjugated porphyrin-based donor-acceptor chromophores possess high first-order hyperpolarizability (*β*_zzz_ ≈ 2500 × 10^−30^ esu at 800 nm in CHCl_3_), and they are 5–10 times more voltage sensitive than **FM4-64** (Reeve et al., 2013, Reeve et al., 2009). One of the major criteria for SHG-based dyes is that they must localize effectively at the plasma membrane of cells and their major transition dipole moment (TDM) should be collinearly oriented with the polarization of laser light to generate high signal (Khadria et al., 2017, Reeve et al., 2012). Dicationic and zwitterionic donor-acceptor porphyrin dyes, **JR-2** and **JR-3** (Figure 1), have been shown to localize in the plasma membranes of live SK-OV-3 cells; however, they require more than 20 mW of laser power (100 fs pulse width; 80 MHz repetition rate) at 10 μM concentration for SHG imaging (Reeve et al., 2009). Such a high laser power is not suitable for live cell imaging. We later discovered that the plasma membrane localization of **JR-2** and **JR-3** dyes is not reproducible in other cell types, and the dyes are internalized by the cells in 5–10 min after incubation, as discussed in this article. To develop a highly SHG-efficient porphyrin-based dye with robust plasma membrane localization, we synthesized a range of amphiphilic dyes with different hydrophilic head groups and studied their behavior in live cells. We categorized the amphiphilic dyes in three classes based on the hydrophilic head groups: (1) cationic, (2) zwitterionic, and (3) non-charged. Based on the results from cellular studies of dyes with the different hydrophilic head groups, we designed and synthesized a new tricationic donor-acceptor-based porphyrin dye that localizes effectively in the plasma membrane of cells to give bright SHG signals at low laser powers (≤5 mW). We demonstrated the SHG and TPEF-based multimodal imaging of the tricationic dye with commercial cellular organelle trackers in the conventional green and red light regions, which are frequently used in fluorescence microscopy. The porphyrin-based dyes emit at wavelengths greater than 630 nm, and they do not give any background signal in the conventional green and red regions. To quench its fluorescence, we synthesized the copper(II) complex of the tricationic dye and demonstrated its plasma membrane localization in HEK293T cells. Here we present the synthesis of six new amphiphilic porphyrin dyes and investigate their use in multiphoton imaging of live cells along with other porphyrin dyes (Reeve et al., 2009).

## Results and Discussion

### Synthesis

**Scheme 1:**
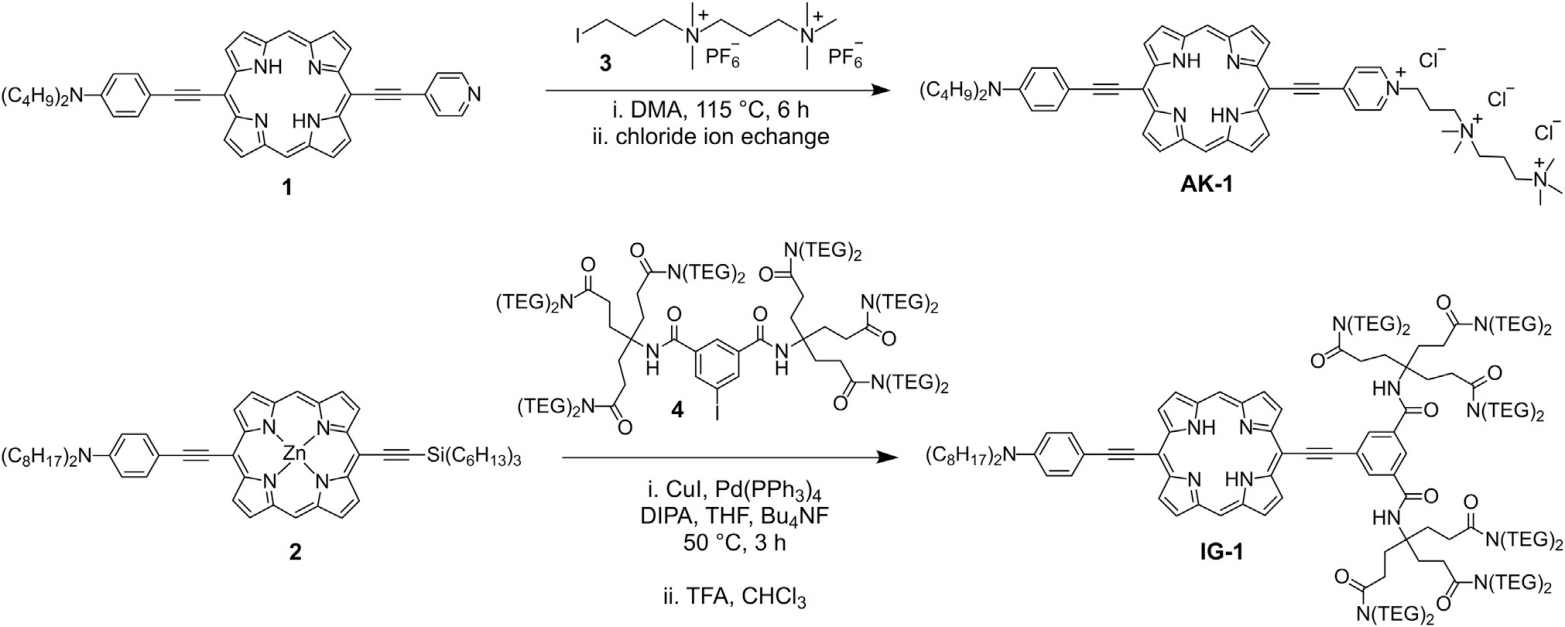
Synthesis of Tricationic Porphyrin Dye **AK-1** and Non-Charged Amphiphilic Porphyrin Dye **IG-1**

We have synthesized several far-red to near-infrared (NIR) light absorbing and emitting amphiphilic porphyrin dyes functionalized with different hydrophilic head groups (Figures 1 and S1), such as dications, zwitterions, and non-charged ethylene glycols. We synthesized the dicationic and zwitterionic dyes **JR-2** and **JR-3** as previously reported (Reeve et al., 2009). Dyes **JR-2** and **JR-3** have been reported to stain the plasma membrane of SK-OV-3 cells; however, we later found that the plasma membrane localization was not observed in other cell types, such as HEK 293T, LN-18, and rat hippocampal cultured neurons. The dyes are internalized by these cells in less than 10 min, perhaps owing to the imbalance between the hydrophilicity and hydrophobicity of the dyes (Barsu et al., 2010). Like **JR-2** and **JR-3**, the commercial SHG dyes **FM4-64** and **di-4-ANEPPS** are dicationic and zwitterionic, respectively (Figure 1); however, they localize in the plasma membrane of live cultured cells (Bolte et al., 2004, Dombeck et al., 2005, Millard et al., 2003, Preuss and Stein, 2013). Since the lengths of the porphyrin-based dyes are almost twice that of **FM4-64** and **di-4-ANEPPS**, the degrees of their hydrophobicity and hydrophilicity are not balanced for effective plasma membrane localization. We synthesized new porphyrin dyes, **JF-1**, **JF-2**, **JW-1**, and **IG-1**, with enhanced hydrophilicity (Figure 1). **JF-1** and **JF-2** are more hydrophilic than **JR-2** and **JR-3** because of the presence of extra triethylene glycol (TEG)-substituted aryl groups attached at the meso positions of the porphyrins. The complete procedures for the synthesis of **JF-1** and **JF-2** are given in the Supplemental Information. The tricationic porphyrin dye **AK-1** and the neutral dyes **IG-1** and **JW-1** were synthesized from porphyrins **1** and **2**, respectively (Scheme 1). While synthesizing **AK-1**, we found that the reaction completes successfully in dimethylacetamide (DMA); however, if the alkylation is performed in other solvents such as dimethylformamide (DMF), decomposition predominates. To the best of our knowledge, this is the first example of an isolated linear tricationic porphyrin-based amphiphilic dye. **AK-1.Cu** was synthesized by treating **AK-1** with copper(II) acetate. Neutral amphiphilic dye **IG-1** was synthesized by *in situ* removal of the trihexylsilyl group of **2** using tetrabutylammonium fluoride and Sonogashira coupling with **4** followed by removal of zinc with TFA (Scheme 1). Porphyrin **JW-1** was prepared similarly using the hexaethylene glycol (HEG)-substituted iodoisophthalic acid instead of **4**. **JW-1** and **IG-1** dyes were functionalized with isophthalic derivatives substituted with four HEG and twelve TEG groups, respectively, instead of the pyridinium-based electron-acceptor group as the hydrophilic moiety. The dyes do not require pyridinium-based electron-acceptor groups because it has been previously shown that an acceptor group does not substantially contribute toward the nonlinear optical properties of free-base donor-acceptor-substituted porphyrin dyes (Annoni et al., 2005, Lopez-Duarte et al., 2013, Morotti et al., 2006). Multiple HEG and TEG groups were used to enhance the aqueous solubility and amphiphilicity of dyes for efficient plasma membrane localization.

**Figure 2:**
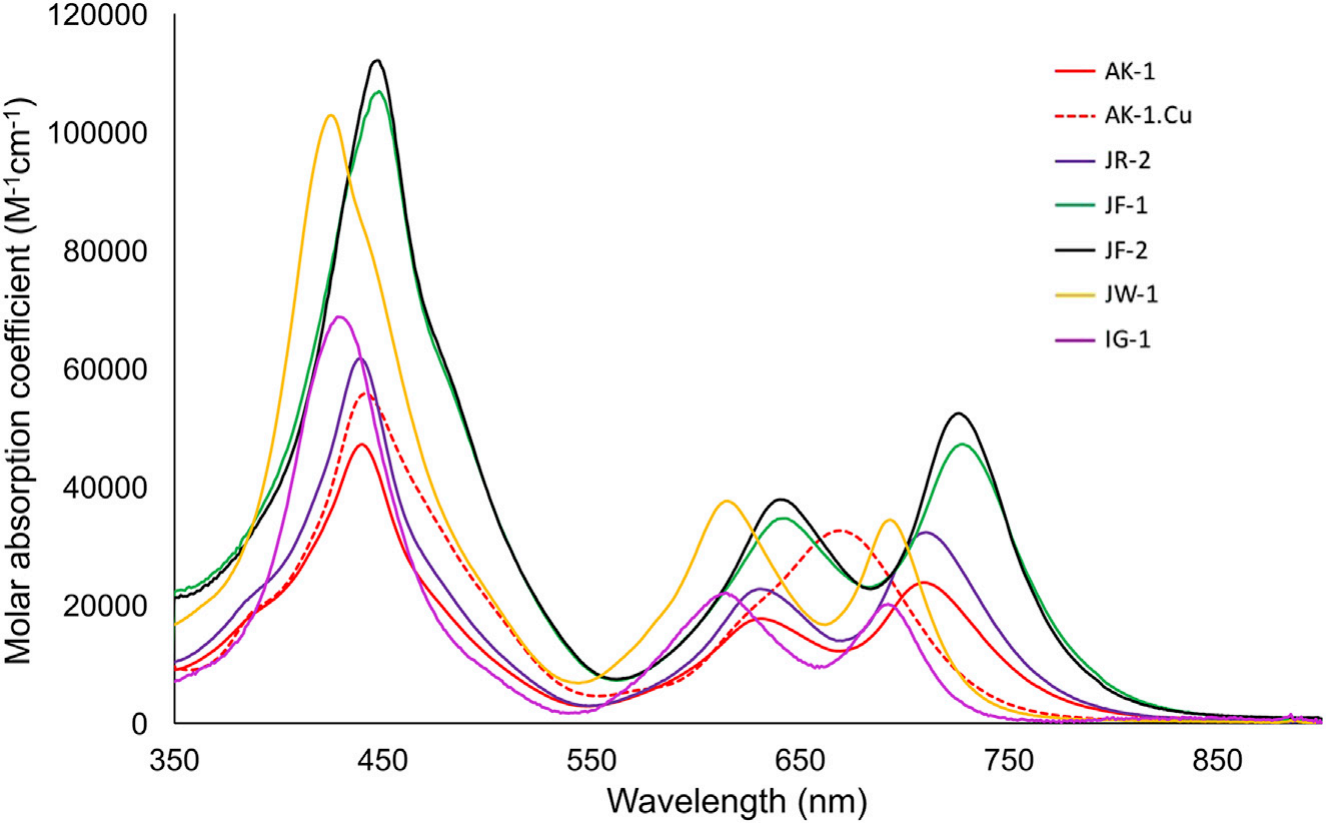
UV-Visible Absorption Coefficient Spectra of the Dyes Measured in DMF

All the porphyrin-based dyes, **AK-1**, **AK-1.Cu**, **JR-2**, **JR-3**, **JF-1**, **JF-2**, **JW-1**, and **IG-1**, have similar absorption spectra (Figure 2) with low fluorescence quantum yields (<0.01 in DMF). The non-charged amphiphilic dyes **JW-1** and **IG-1** stain the intracellular area to give only TPEF signals (Figure S2). Despite possessing large hydrophilic groups, these dyes cross the cell membrane.

### Cell Imaging

The cellular localization of all the dyes was studied in HEK293T cells. These cells were chosen because they can be easily cultured and are widely used in biological studies. The dyes were incubated in the cells for 3–5 min at a concentration of 20 μM (unless otherwise specified) at 20°C in Hank’s balanced salt solution (HBSS) buffer. The incubated cells were imaged under the microscope at 870 nm using up to 5 mW laser power (measured at the sample; 70 fs pulse width; 80 MHz repetition rate).

**Figure 3:**
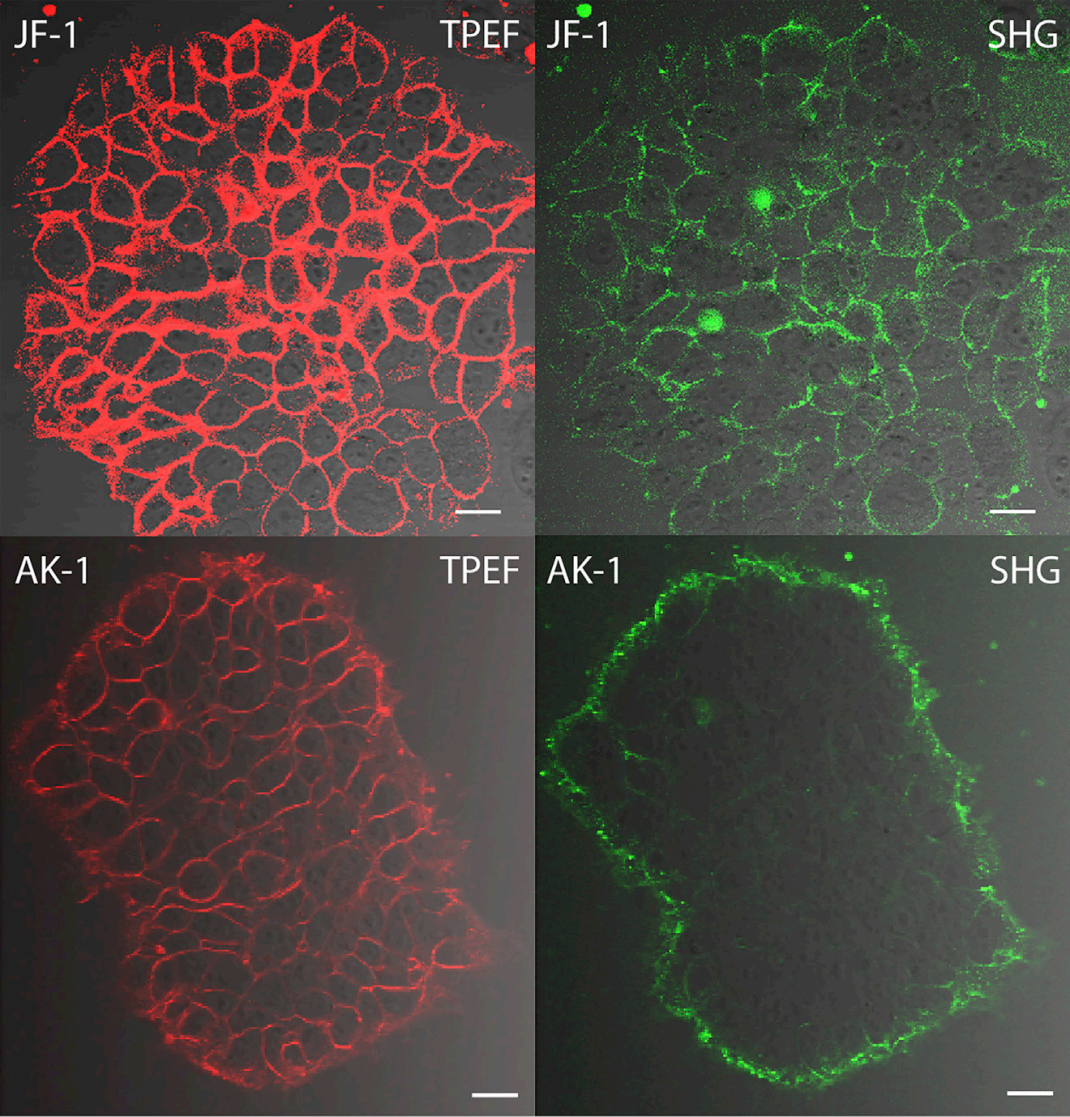
Cellular Imaging of **JF-1** and **AK-1** **JF-1** (10 μM at 20 mW laser power) and **AK-1** (20 μM at 5 mW laser power) localize in the plasma membrane of HEK293T cells to generate both fluorescence and SHG signals. The images of **JF-1** are digitally enhanced for clarity. No SHG can be seen from individual cells in the case of **AK-1**; this is attributed to the centrosymmetric arrangement of dyes where the plasma membranes of the cells touch each other. λ_ext_ = 840 nm (**JF-1**), 870 nm (**AK-1**). The images are overlays of TPEF/SHG and transmitted images. Scale bar, 20 μm.

**Figure 4:**
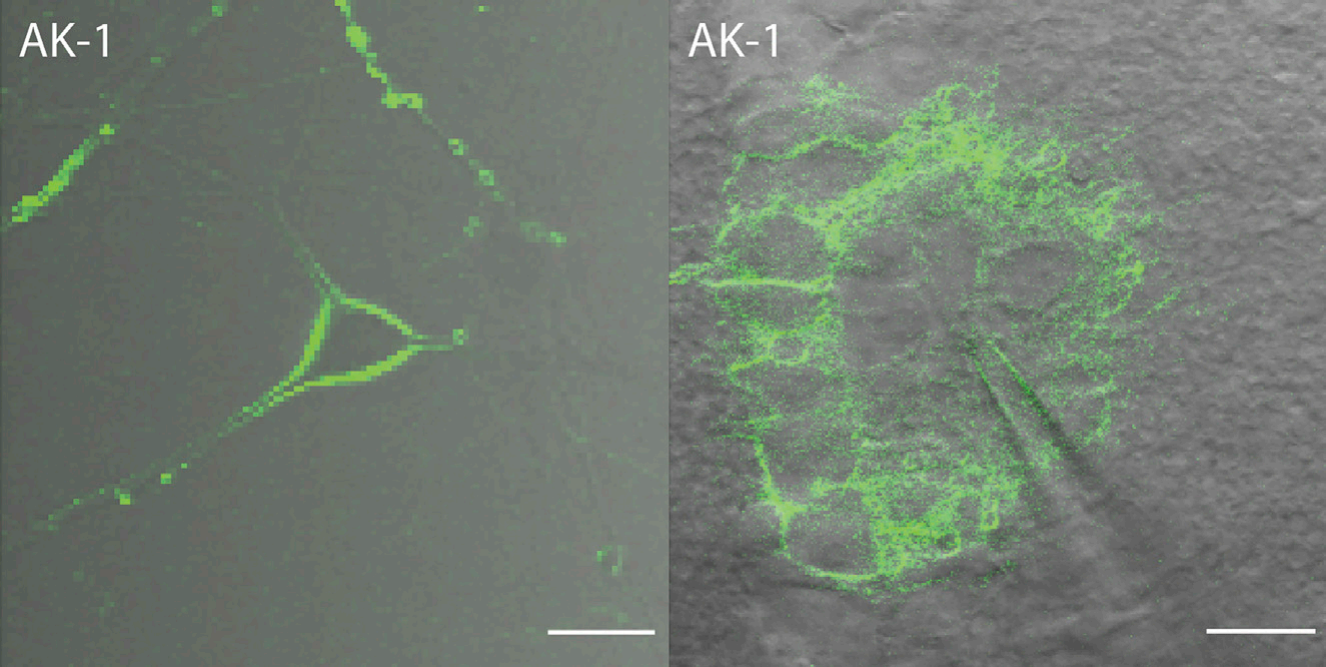
Neuronal SHG Imaging of **AK-1** SHG images of **AK-1** from the plasma membrane of cultured rat hippocampal neurons (40 μM) and the neurons deeply located in *ex vivo* acute mice brain slices (25 μM). In cultured neurons, the dye was incubated in the bath, whereas in mice brain slices, the dye was injected using a micropipette. Scale bar, 20 μm.

The positively charged dicationic dyes **JF-1** and **JR-2** localize at the plasma membrane of HEK293T cells (Figures 3 and S3, respectively). However, the plasma membrane localization of **JR-2** is not effective, and it is internalized by the cells within a few minutes after incubation, whereas **JF-1** remains localized for more than 2 hr. After **JR-2** is internalized by the cells, SHG signals are visible from the intracellular organelles. The organelles giving SHG signals have the shape of semi-concentric circles attached to the nucleus, suggesting that they are ER (Figure S3) (Fawcett, 1981, Goyal and Blackstone, 2013). Co-localization experiments with BodipyTR-based ER Tracker Red dye confirm that the dye localized at the endoplasmic reticula (Figure S4) along with other cellular organelles. Cationic FM dyes, such as **FM4-64**, are widely used as fluorescent endocytosis markers and have been used for vesicle trafficking and found to stain several cell organelle membranes (Betz et al., 1996, Bolte et al., 2004, Fischer-Parton et al., 2000, Gaffield and Betz, 2006, Hickey et al., 2002). Hence, it is not surprising that the dicationic dye **JR-2** stains the ER non-centrosymmetrically to give SHG signals. This is the first time that an SHG image has been seen from a dye labeling intracellular organelles. Previously, aggregates of pyropheophorbide-a formed within lipid nanoparticles have been shown to generate SHG signals from the intracellular area but the pyropheophorbide-a did not directly stain the intracellular organelles (Cui et al., 2015). On the other hand, **JF-1** does not cross the cell membrane and gives SHG signals from the plasma membrane (Figures 3 and S5). The only structural difference between these two dyes is that **JF-1** is functionalized with hydrophilic TEG-substituted aryl groups at the meso positions of the porphyrin core, making it more hydrophilic. However, the intensity of SHG signals from **JF-1** is low at 10 μM dye concentration even at 20 mW of laser power. Higher laser power results in cell death within a few minutes. Increasing the concentration of the dye beyond 25–30 μM (in 0.1% DMSO as solubilizing agent) leads to aggregation and does not improve the brightness of SHG. We postulate that the reason for low SHG signal could be dual: (1) the uptake of the dye in the plasma membrane of the cells is limited by the TEG-substituted aryl groups located at the meso positions of porphyrin, resulting in overall reduced fluorescence and SHG signals or (2) the TDM of the dye is not well aligned perpendicular to the plane of the membrane (Khadria et al., 2017, Reeve et al., 2012). To test these two points, we removed the TEG-substituted aryl groups from the meso positions of porphyrins, increased the number of cationic charges in the hydrophilic head group to three, and substituted the octyl chains at the aniline-based donor group with butyl chains to synthesize a new tricationic dye, **AK-1**. The new dye, **AK-1**, is more hydrophilic than **JR-2** and **JF-1** but has a similar donor-porphyrin-acceptor structure. While testing the localization of **AK-1** in cells, we found that it effectively localizes at the plasma membrane of cells for more than 2 hr to give brighter SHG signals than **JF-1** at similar imaging conditions (Figure 3). SHG signals cannot be seen from the individual cells stained with **AK-1** in Figure 3, perhaps because the dyes are centrosymmetrically arranged where the plasma membranes of the cells touch each other. Apart from SHG, the TPEF images captured using **AK-1** are also brighter than those captured using **JF-1**, suggesting that the TEG-substituted aryl groups hinder effective plasma membrane localization. This result also consolidates our initial assumption that hydrophobicity and hydrophilicity of a dye must be balanced for effective plasma membrane localization. The new tricationic dye, **AK-1**, also gave bright SHG signals from cultured rat hippocampal neurons and the neurons located deep (50–100 μm) in acute mouse brain slices (Figure 4). In the cultured neurons, dye concentration up to 40 μM was used to reduce the laser power to 1 mW. In mouse brain slices, only 25 μM of dye was used. Dombeck et al. reported SHG signals from rat brain slices by injecting up to 500 μM of **FM4-64**; however, they also used a scavenger, Advasep, to remove the dye that gets absorbed into the neural tissue (Dombeck et al., 2005, Kay et al., 1999). Without use of a scavenger, **FM4-64** is absorbed all over the slices, resulting in significant background signals (Figure S6). **AK-1** generates a good SHG signal at one-twentieth of the concentration of **FM4-64** without needing a scavenger. We performed the imaging up to 30 min after pressure injection of **AK-1** in slices and did not observe any loss of signals due to dye flip-flop.

### Multimodal Imaging

**Figure 5:**
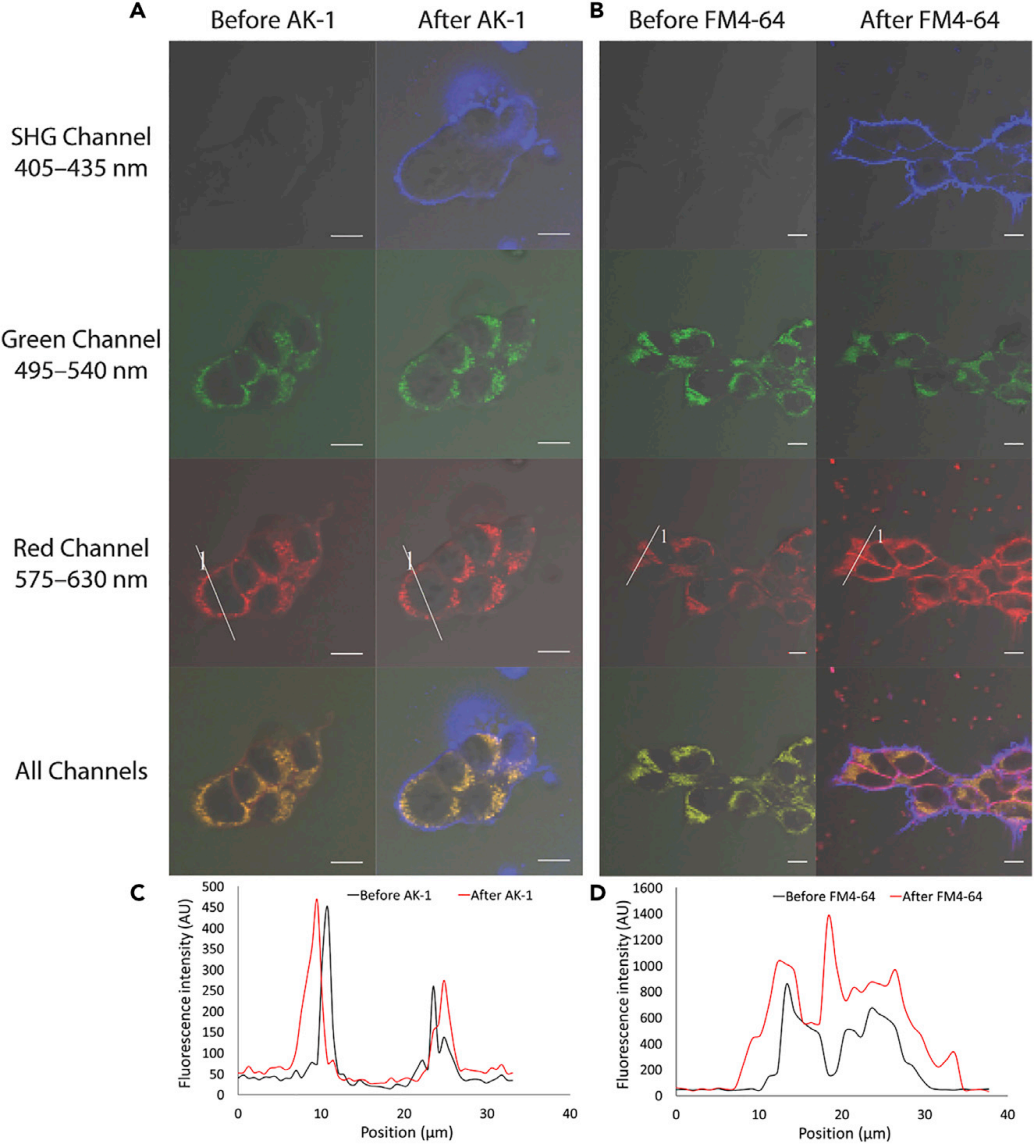
Comparison of Multimodal Imaging of **AK-1** with **FM4-64** HEK293T cells were incubated with **RH123** and LysoTracker Yellow HCK-123 dyes. Images were taken by photon counting before and after the addition of **AK-1** (A) or **FM4-64** (B). The sizes of the cells had expanded by ∼2 μm when they were imaged the second time, after the addition of **AK-1** or **FM4-64**. The SHG channels clearly show that SHG is generated from the plasma membrane of the cells after addition of **AK-1** and **FM4-64**. In the green channels, no significant changes in the signals were observed after the addition of either **AK-1** or **FM4-64**. In the red channel, there was no change in the fluorescence signal after the addition of **AK-1**, as shown in the intensity profile (C) of the area depicted by the line (called 1) drawn across a cell. However, after the addition of **FM4-64**, there was a significant increase in the fluorescence signal from the intracellular area and the plasma membrane (across the line 1 as shown in D) as reported (Nuriya et al., 2016). Merged images of all the channels show substantial difference in the fluorescence signals from the intracellular area before and after the addition of **FM4-64**, whereas the difference in the signal generated from the intracellular area before and after addition of **AK-1** is negligible. Scale bar, 10 μm.

Multimodal imaging harnesses the advantages of several imaging techniques to visualize discrete biological processes simultaneously, which otherwise would not be possible by using just one technique at a time (Awasthi et al., 2016, Cheng et al., 2011, Nuriya et al., 2016, Weissleder and Pittet, 2008). TPEF and SHG-based multimodal imaging is mostly restricted to the situation where part of the sample itself generates SHG signals, for example, sarcomeres in cardiomyocytes, thus requiring only a single dye to be used for fluorescence (Awasthi et al., 2016). We performed TPEF and SHG-based multimodal imaging of far-red to NIR emitting dye **AK-1** in HEK293T cells with two fluorescent cell trackers, mitochondrial tracker **RH123** and LysoTracker Yellow HCK-123 (Figure 5). HEK293T cells were stained with both commercial fluorescent trackers and imaged before and after the addition of **AK-1**. Although **AK-1** generates strong SHG signals from the plasma membrane, it does not give any fluorescence signals or interfere with those of the commercial fluorescent trackers in the green (495–540 nm) and red (570–625 nm) regions. This is because **AK-1** emits fluorescence at wavelengths greater than 630 nm (Figure S1) with a low fluorescence quantum yield (<0.01). In contrast to **AK-1**, the commonly used plasma-membrane-bound styryl SHG dye, **FM4-64**, emits a strong fluorescence signal from the plasma membrane as well as from the intracellular area in the red region, thus contaminating the fluorescence from the commercial trackers. Until now, there has been only one report of an SHG-only dye (named as **Ap3**) that is suitable for multimodal imaging (Nuriya et al., 2016). Although **Ap3** possesses negligible fluorescence quantum yield and does not emit any fluorescence even in the far-red region unlike **AK-1**, it generates similar or less SHG signals even than **FM4-64** in contrast to the donor-acceptor porphyrin-based **AK-1**, which gives almost three times more SHG signal than **FM4-64** (Khadria et al., 2017, Lopez-Duarte et al., 2013, Nuriya et al., 2016, Reeve et al., 2009). Although **AK-1** gives a fluorescence signal in the far-red to NIR regions even with a low fluorescence quantum yield, it does not give any fluorescence in the green and red regions, where most of the commercial cell markers emit (Bestvater et al., 2002). This makes **AK-1** a very potent candidate for TPEF and SHG-based multimodal imaging.

**Figure 6:**
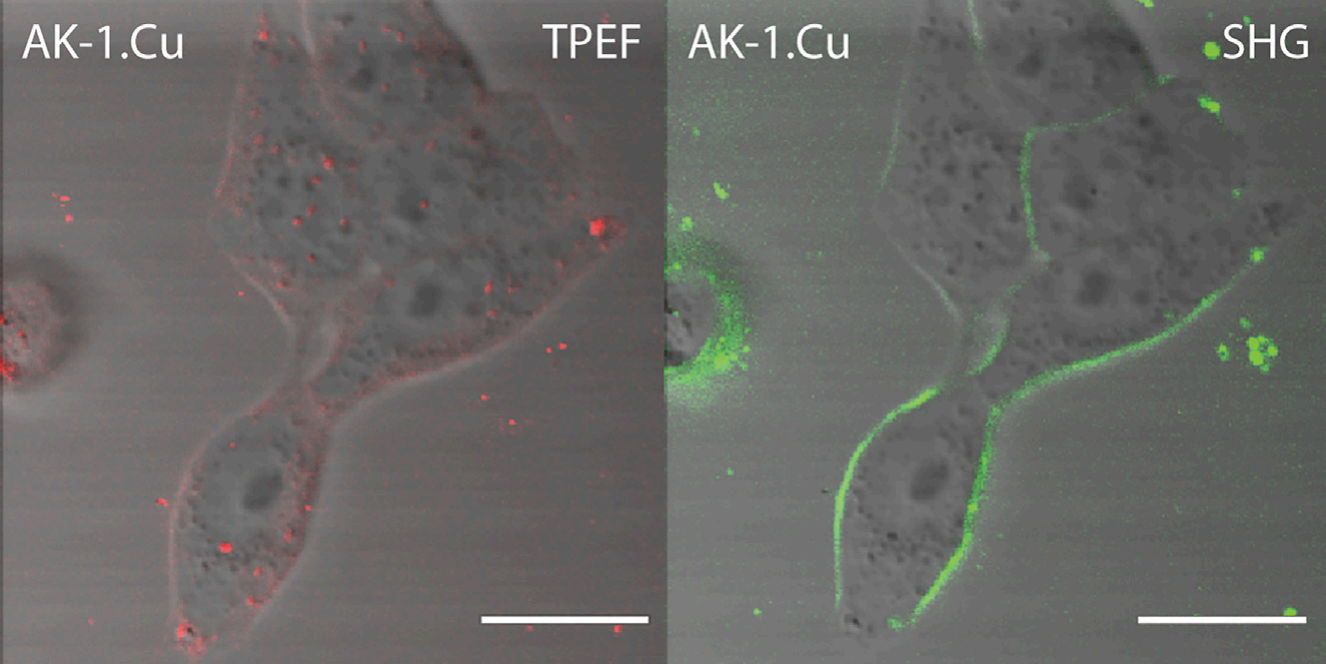
TPEF and SHG Images of HEK 293T Cells Incubated with the Copper(II) Complex of **AK-1** No fluorescence is seen from the dye localized at the plasma membranes of the cells, whereas significant SHG signals are visible. Scale bar, 20 μm.

Fluorescent dyes are often associated with problems of photobleaching, which may be avoided by dyes that give only SHG. We synthesized the copper(II) complex of **AK-1** so that it works as an SHG-only dye, without any collateral fluorescence. Copper(II) and nickel(II) cations are known to quench the fluorescence of porphyrins without generating singlet oxygen and, hence, phototoxicity (Kim et al., 1984, McCarthy and Weissleder, 2007, Redmond and Gamlin, 1999). Previously, we have reported that apart from the free base, the copper(II) and nickel(II) complexes of donor-acceptor porphyrins possess SHG efficiency (Reeve et al., 2009). However, compared with free-base porphyrins, the SHG efficiency of the copper(II) complex of the donor-acceptor porphyrin is reduced almost by half, whereas that of the nickel(II) complex of the donor-acceptor porphyrin is reduced by more than ten times at 840 nm in DMF (Reeve et al., 2009). As expected, the copper(II) complex of **AK-1** did not give fluorescence in the NIR region but gave bright SHG from the plasma membrane of HEK293T cells (Figure 6). We also synthesized the copper(II) complex of **JF-1**, which behaved in a manner similar to that of the copper(II) complex of **AK-1** (Figure S7) to give only SHG signals from the plasma membrane of the cells.

**Figure 7:**
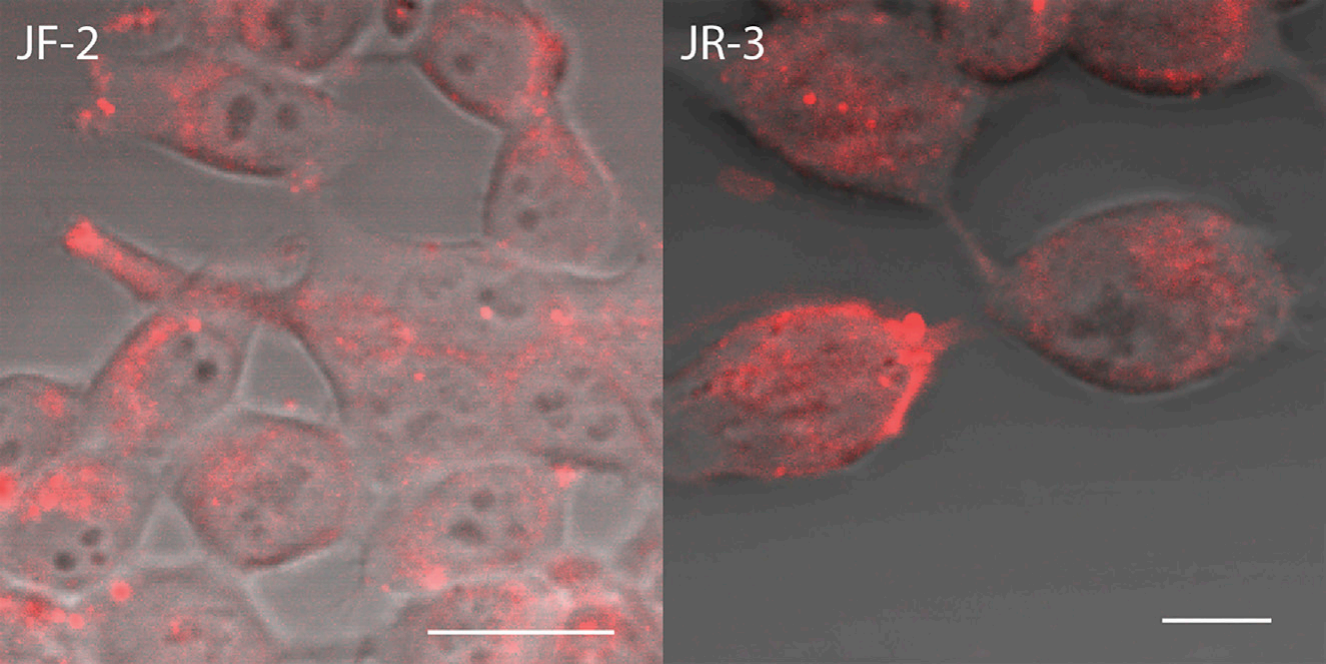
TPEF Images of Cells Incubated with **JF-2** and **JR-3** Dyes The dyes show no plasma membrane localization. No SHG signals were observed either from the plasma membrane or the intracellular area. Scale bar, 20 μm.

On testing the zwitterionic dyes **JR-3** and **JF-2**, both entered the cells without any plasma membrane localization (Figure 7). Given that the dicationic dye **JF-1**, an analogue of **JF-2**, localized effectively at the plasma membrane, it was expected that **JF-2** too will localize at the plasma membrane. It appears that the zwitterionic sulfonate dyes localize less efficiently in the plasma membrane than the cationic dyes perhaps because of the decreased hydrophilicity of the zwitterions compared with cations. To test this idea, we compared the cellular localization of the commercial dicationic dye **FM4-64** with that of the zwitterionic dye **di-4-ANEPPS**. Both the dyes gave SHG signals from the plasma membrane of HEK293T cells; however, **di-4-ANEPPS** also gave fluorescence from inside the cells, whereas **FM4-64** gave minimal fluorescence from the intracellular area when imaged within a few minutes after staining the cells (Figure S8). This result suggests that the hydrophilicity of a molecule plays a significant role in the plasma membrane localization of dye. It is well established that for plasma membrane localization, the dyes should be lipophilic and longer hydrophobic alkyl chains ensure irreversible localization (Betz et al., 1996, Horan et al., 1990); however, the role of hydrophilicity has not been thoroughly investigated. Previously, it has been observed that the dicationic version of the amphiphilic dye, ANNINE-6plus, ensures better plasma membrane binding than the zwitterionic version, ANNINE-6, but the importance of hydrophilic head groups of amphiphilic dyes in plasma membrane binding was not studied (Fromherz et al., 2008). Our results show that the dyes must be sufficiently hydrophilic for plasma membrane localization.

### Conclusion

We have synthesized a library of far-red to NIR light absorbing and emitting donor-acceptor-based porphyrin dyes with different live cell localization properties depending on the type of hydrophilic head groups. Of cationic, zwitterionic, and non-charged hydrophilic head groups, we found that the cationic porphyrin dyes have the highest affinity toward the plasma membrane. Although fluorescence generally gives brighter images than SHG, the porphyrin dyes reported here generate comparable or better SHG images. The tricationic dye **AK-1** localizes at the plasma membrane of live cells to give bright SHG signals at less than 5 mW of laser power. The far-red to NIR fluorescence and high SHG efficacy of **AK-1** make it suitable for TPEF and SHG-based multimodal imaging in combination with commercial fluorescent cell markers. The dye also gives bright SHG signals from *ex vivo* neurons located 50–100 μm deep inside acute mice brain slices. The photostable copper(II) complexes of **AK-1** and **JF-1** are the second examples of SHG-based dyes reported so far that give negligible TPEF and the first for porphyrin-based dyes. Although the aqueous compatible neutral porphyrin-based dyes **JF-1** and **IG-1** do not generate SHG in live cells, they are potential candidates for photodynamic therapy (PDT) because free-base porphyrins are known to generate singlet oxygen for PDT (Balaz et al., 2009, Kuimova et al., 2009, Pawlicki et al., 2009). Apart from newly synthesized dyes, we also discovered that one of our previously reported dyes, **JR-2**, stains intracellular organelles to give to SHG signals. Here, we present several highly SHG-efficient probes that localize reliably in cellular membranes to give SHG at low laser powers and that are suitable for deep imaging and TPEF/SHG-based multimodal imaging.

## Methods

All methods can be found in the accompanying Transparent Methods supplemental file.

## Supplemental Information

Document S1. Transparent Methods, Figures S1–S13, and Schemes S1–S4
