# Supplementary material for: Porphyrin Dyes for Nonlinear Optical Imaging of Live Cells
Source: iScience. 2018 May 26;4:153–63. doi: 10.1016/j.isci.2018.05.015 (PMC6147020; doi:10.1016/j.isci.2018.05.015)
Supplement: Document S1. Transparent Methods, Figures S1–S13, and Schemes S1–S4 [file mmc1.pdf]

ISCI, Volume *4*

Supplemental Information

Porphyrin Dyes for Nonlinear Optical Imaging of Live Cells

Anjul Khadria, Jan Fleischhauer, Igor Boczarow, James D. Wilkinson, Michael M. Kohl, and Harry L. Anderson

Supplemental Data Items

1. Linear optical spectra of the compounds

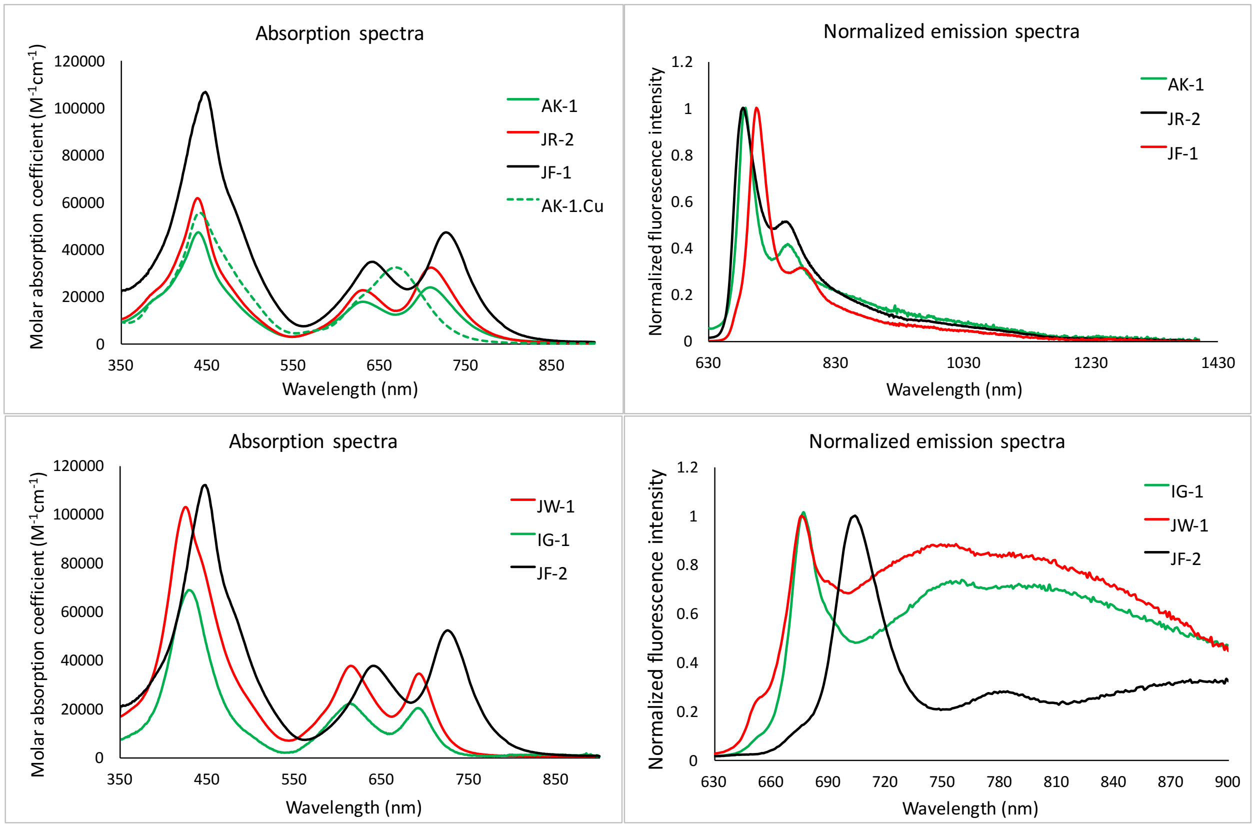

Figure S1. Linear optical spectra of the porphyrin compounds, related to Figure 2: Comparison of absorption and emission spectra (in DMF at 25 °C) of cationic charged dyes **AK-1**, **JR-2**, **JF-1**, **AK-1.Cu** and neutral dyes, **JW-1**, **IG-1**, and **JF-2**.

2. Cell imaging

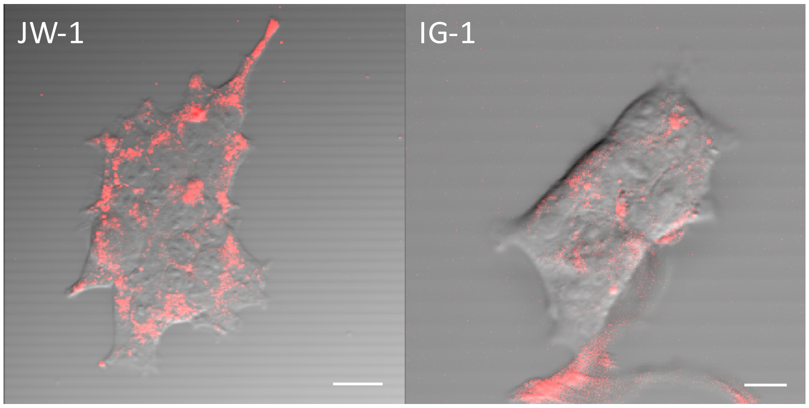

Figure S2. Imaging of non-charged dyes JW-1 and IG-1 in HEK 293T cells, related to Figure 3: The fluorescence images of the dyes, **JW-1** and **IG-1** in the cells show no localization in the plasma membrane. No SHG was seen from the intracellular area. Scale = 20 μm (**JW-1**), 10 μm (**IG-1**).

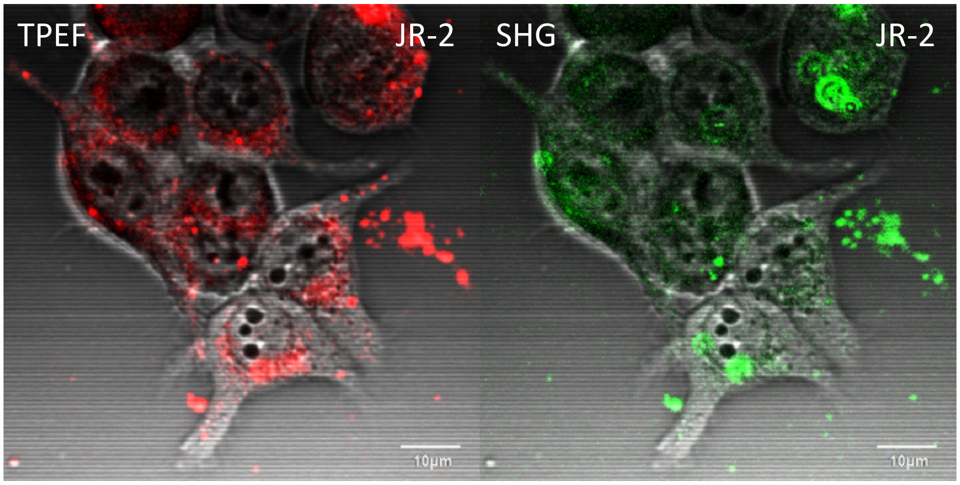

Figure S3. Imaging of JR-2 in HEK 293T cells, related to Figure 3: The fluorescence and SHG **JR-2** in HEK 293T cells. The dyes could be seen staining the intracellular organelles of the cells to give both fluorescence and SHG signals. λ_{ext} = 840 nm, scale bar = 10 μm.

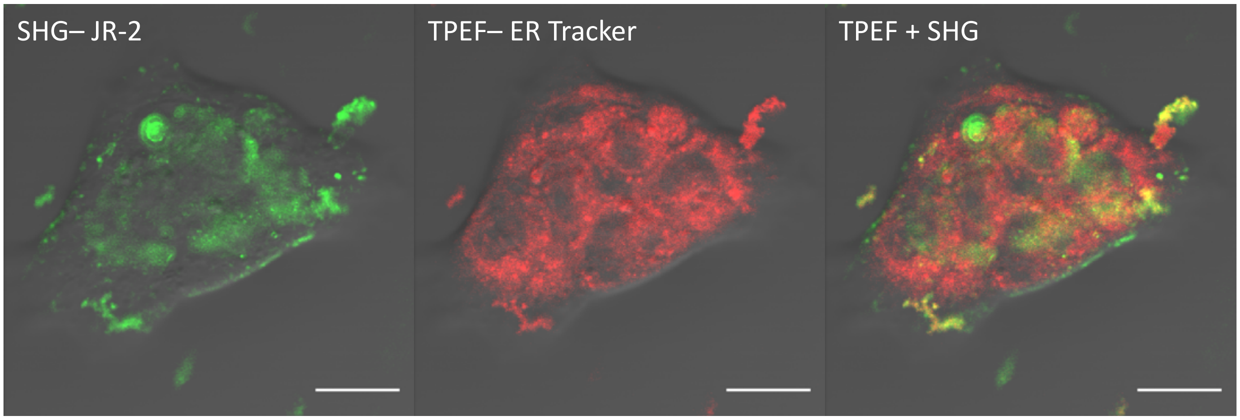

Figure S4. Co-localization of JR-2 with ER Tracker in HEK 293T cells, related to Figure 3: The SHG image is from only **JR-2**, while the fluorescence image is from only ER-Tracker™ Red dye detected in the red channel (570–625 nm). Fluorescence + SHG shows the co-localization of **JR-2** with ER-Tracker™ Red dye. Scale bar = 20 μm.

The co-localization experiment (Figure S4) shows that SHG is generated from **JR-2** dye molecules staining the intracellular organelles including endoplasmic reticulum. Although the porphyrin dye also emits fluorescence, it is not detected because the light was passed through a 570–625 nm filter (the dye does not emit in this range) before being detected through the PMT.

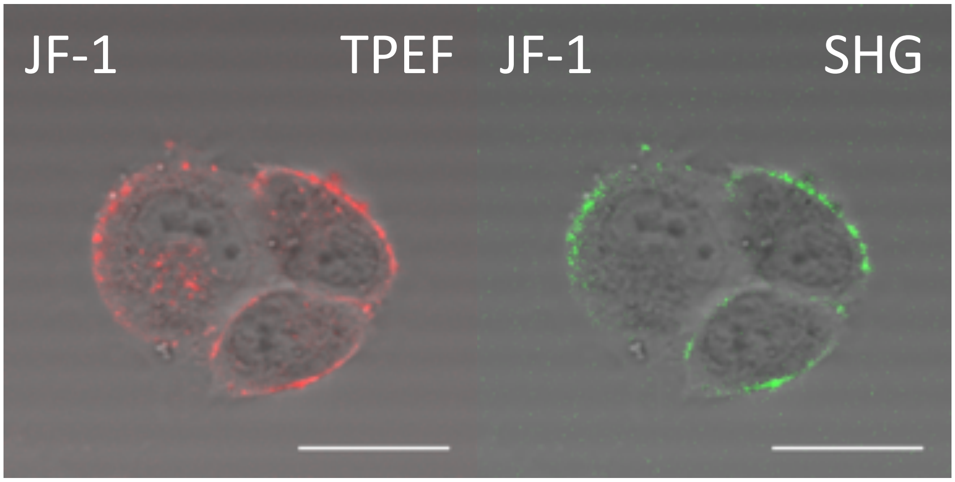

Figure S5. Imaging of JF-1 in LN-18 cells, related to Figure 3: Fluorescence and SHG images of **JF-1** (10 µM) in LN-18 cells. λ_{ext} = 840 nm, scale bar = 20 µm.

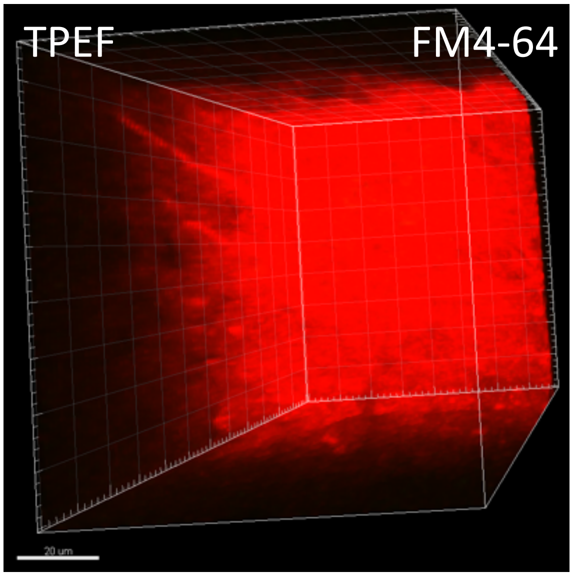

Figure S6. Fluorescence imaging of FM4-64 in mouse brain slice, related to Figure 4: 3D image of a section of mouse brain slice stained with **FM4-64** (50 µM) without Advasep. The dye could be seen absorbed all over the area staining the neural tissue and cells alike. Scale bar = 20 µm.

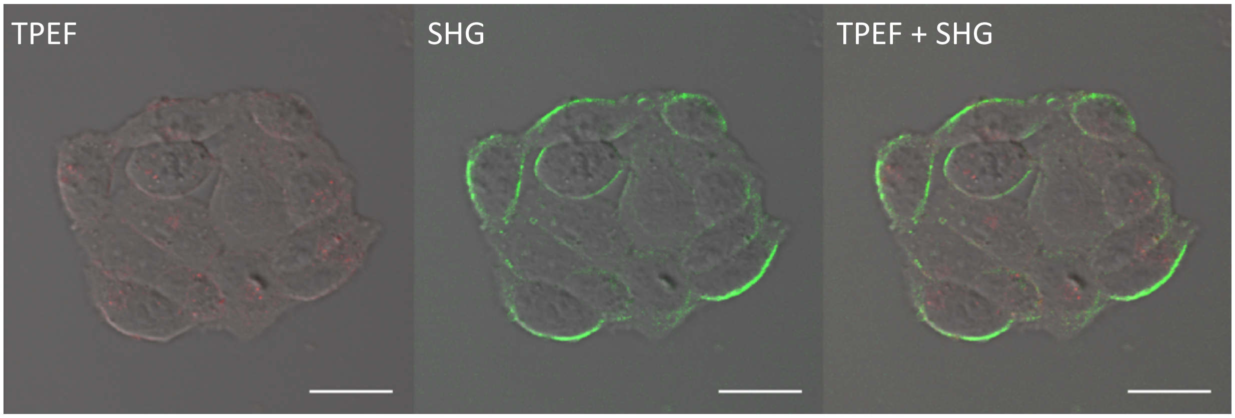

Figure S7. SHG imaging of only SHG dye, JF-1.Cu, related to Figure 6: Images of **JF-1.Cu** (40 µM) incubated in LN-18 cells. The LN-18 cells were cultured and maintained following the same protocol as HEK 293T cells. The dye does not emit any fluorescence but generates strong SHG signals from the plasma membrane. The overlay of fluorescence and SHG images show that no yellow color (if red and green are mixed) is generated. λ_{ext} = 850 nm, scale bar = 20 µm.

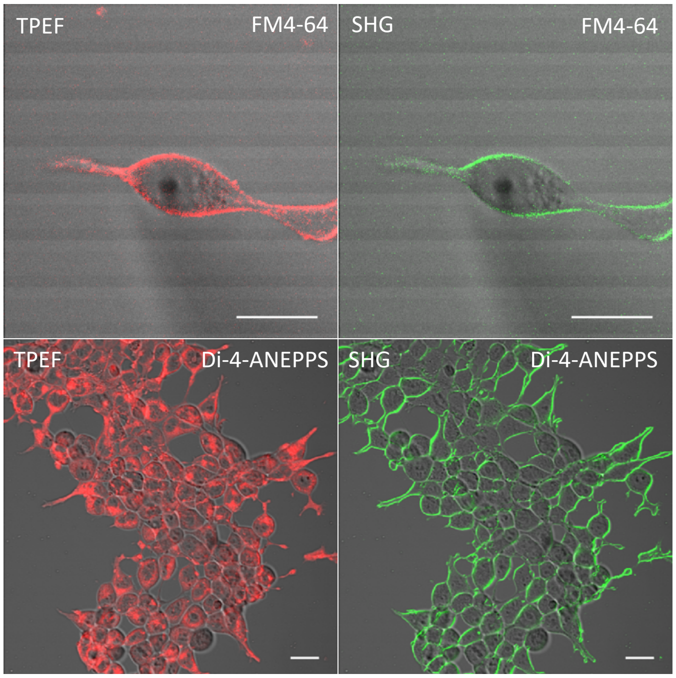

Figure S8. Imaging of FM4-64 and Di-4-ANEPPS as control experiments, related to Figure 7: Dicationic and zwitterionic dyes, **FM4-64** (10 µM) and **di-4-ANEPPS** (10 µM) incubated in with the HEK 293T cells. The images were taken immediately after the dye incubation. **FM4-64** does not get internalized in the cells just after incubation as minimal fluorescence is visible from the intracellular area. **Di-4-ANEPPS** is internalized by the cells apart from staining the plasma membrane. Significant fluorescence is seen from inside the cells stained with **di-4-ANEPPS** apart from bright SHG from the plasma membrane. λ_{ext} = 840 nm, scale bar = 20 µm.

Transparent Methods

1. Linear optical properties of the porphyrin-based dyes

The UV-Vis (Perkin Elmer Lambda 20) and fluorescence (Edinburgh Instruments, Spectrofluorometer FS5) measurements were performed in DMF at 25 °C.

Measurement of fluorescence quantum yields

The quantum yield of a compound is given by the equation:

$$\phi_C = \phi_R \frac{I_c A_R n^2}{I_R A_C n_{\mathrm{ref}}^2}$$

where ϕ_C is the quantum yield of the compound, ϕ_R is the quantum yield of the reference compound, I_c is the fluorescence intensity of the compound, I_R is the fluorescence intensity of the reference compound, A_c is the absorbance of the compound (<0.1), A_R is the absorbance of the reference (<0.1), n is the refractive index of the solvent (DMF = 1.4305) in which the compound of interest is dissolved and n_{ref} (CH_2Cl_2 = 1.4244) is the refractive index of the solvent in which the reference is dissolved. The absorbance values, A_c and A_R were measured at the same wavelengths at which the emission of the compounds was measured. To quantify the fluorescence intensities, I_c and I_R, the emission spectra of the compounds were integrated over the whole region. The reference was analyzed in CH_2Cl_2 while the unknown compound was analyzed in DMF. The quantum yields of the dyes were calculated by measuring their absorbances and fluorescence intensities and then comparing them with the absorbance and fluorescence intensity of the reference compound, pyropheophorbide-a methyl ester according to the above equation. For each compound, five measurements were done at different absorbances (<0.1). The reported quantum yield of pyropheophorbide-*a* methyl ester (ϕ = 0.22 in CH_2Cl_2) was used as a reference (Sasaki et al., 2010).

2. Cell Imaging

Culturing HEK 293T cells: A stock of human embryonic kidney (HEK) 293T cells was procured from ATCC (American Type Culture Collection) company. All the media and supplements were procured from Sigma Aldrich unless otherwise specified. The cells were suspended in 10 mL of phenol red free DMEM media (FluoroBrite™ from ThermoFisher Scientific) containing 4.5 g/L glucose, supplemented with 10% fetal bovine serum (FBS), 2 mM L-glutamine and 1 mM sodium pyruvate. The cell suspension was centrifuged at 200 G for 10 minutes to pellet the cells. The supernatant was discarded, and the cell pellet was suspended in 5 mL of phenol-red free supplemented DMEM media and then mixed with 10 mL of the media in a T75 flask and incubated at 37 °C in 5% CO_2 for 48 h. After 48 h, 1/10th of the cells were passaged to a new T75 flask with 15 mL of fresh phenol red free supplemented DMEM media to be incubated at 37 °C in a CO_2 incubator until they are 70% confluent. After the cells became 70% confluent, they were further passaged into six T75 flasks (1/6th of cells in each flask) until they are 70% confluent. Stock solutions were prepared from the six T75 flasks of 1 mL each at a density of 1 million cells/mL in 10% DMSO, 20% FBS supplemented DMEM media (phenol red free) and frozen at −80 °C using Mr. Frosty™ cell freezer.

The cells grown in a T25 or T75 flask were washed with Ca^{2+} and Mg^{2+} free Hank's balance salt solution (HBSS) buffer after decanting the media. The cells were then re-suspended in 5 mL of supplemented media. The cell suspension (500 μL) was then mixed with 6 mL of media in a T25 flask and incubated at 37 °C in a CO_2 incubator until they are about 70% confluent.

Incubation of dye: The cells were plated in poly-D-lysine coated 50 mm glass-bottom dishes (MatTek®) at 37 °C in a CO_2 incubator to 70% confluency. When the cells were confluent, they were washed with Ca^{2+} and Mg^{2+} free HBSS buffer and incubated with the desired concentration of dye in 0.1% to 0.5% DMSO in HBSS buffer (with Ca^{2+} and Mg^{2+} ions). For co-localization and control experiments, **FM4-64** was procured from Biotium under the name SynaptoRed C2. LysoTracker™ Yellow HCK-123, rhodamine 123 (**RH123**), **di-4-ANEPPS**, and ER-Tracker™ Red (BODIPY™ TR Glibenclamide) were procured from ThermoFisher Scientific.

Cultured rat hippocampal neurons: Cultured primary rat hippocampal neurons were a kind gift from Prof. Nigel Emptage, Department of Pharmacology at the University of Oxford. All reagents were procured from Invitrogen unless otherwise stated. Hippocampi were dissected from E18 Wistar rat embryos (Charles River Laboratory), dissociated in 0.5 mg/mL trypsin in HBSS for 15 minutes at 37 °C, washed twice in culture medium and gently triturated in culture medium using a briefly fire polished P1000 plastic pipette tip. Dissociated neurons were plated at a density of ~250/mm^2 on poly-D-lysine coated 50 mm glass bottom dishes from MatTek®. After attachment, neurons were incubated in Neurobasal medium supplemented with 2% fetal calf serum (FCS), 2% B27, 1% Glutamax and 1% penicillin/streptomycin. The day after plating, half the medium was changed for Neurobasal supplemented with 2% B27 and 1% Glutamax only; this medium was used for all further feeds. Cultures were maintained in an incubator at 37 °C perfused with 5% CO_2. Cultures were used for experiments at 14–21 days *in vitro* when synapses are mature. All animal work was carried out in accordance with the Animals (Scientific Procedures) Act, 1986 (UK).

Mice brain slices: Postnatal day (P) 14-21 C57BL/6 mice of both sexes were anaesthetized by isoflurane inhalation. The animals were decapitated in accordance with British Home Office regulations. The brain was removed swiftly and stored in ice-cold (0–4 °C) artificial cerebrospinal fluid (NaCl 126 mM, KCl 3 mM, NaH_2PO_4 1.25 mM, $MgSO_4$ 2 mM, $CaCl_2$ 2 mM, $NaHCO_3$ 26 mM, and glucose 10 mM; pH 7.2–7.4; osmolarity 285–300 mOsm L^{-1}) for approx. 10 min (aCSF). aCSF was continuously bubbled with carbogen gas (95% O_2 and 5% CO_2) for at least 30 min before use. A thin section of dorsal surface was cut with a scalpel after separating the hemispheres. The dorsal part of the hemisphere was glued to a microtome pate for cyanoacrylate adhesive. Horizontal slices of entorhinal cortex (300–350 μm thick) were cut with a vibrotome (Leica VT 1000s) in aCSF.

For imaging experiments, the slices were stored in aCSF and bubbled continuously with carbogen using a perfusion setup. For pressure injection delivery, the dye was dissolved in HBSS buffer solution using 0.1% DMSO and delivered using a pulled patch-clamp–based pipette from Harvard Instruments.

Microscope: The imaging experiments were performed using an Olympus FV1200MPE-BX61WI microscope equipped with Mai Tai® eHP DeepSee™ Ti:Sapphire laser (70 fs pulse width, 80 MHz repetition rate, continuously tunable between 690–1040 nm) from Spectra-Physics. The light was focused using a 2 mm working distance 25X multiphoton objective (XLPLN25XWMP2). For TPEF, the reflected light was passed through a 750 nm short pass filter before being passed through a 540 nm long pass (LP) filter or a dichroic mirror separating the light to pass through green (495–540 nm) and red (570–625 nm) band pass filters and then was detected by PMT detectors (Hamamatsu R3896 for green

and Hamamatsu IR sensitive PMT-R10699 for red). For SHG, the light in the transmitted direction was collected through a 0.9 NA air-based condenser and then passed through a band-pass filter (405–435 nm) before being detected through a PMT detector (Hamamatsu R3896). All the images were acquired in analog-integration mode unless otherwise specified. The images were processed using Olympus Fluoview software and Imaris x64 7.7 software. The images presented here are scanned with a pixel dwell time of 2–12.5 μs/pixel at 512 × 512 pixels.

All the images are taken at 870 nm at ≤5 mW laser power unless otherwise specified. The concentration of the dyes are, **AK-1** = 20 μM (HEK 293T cells), 40 μM (cultured neurons), 25 μM (rat brain slices), **JF-1** = 10 μM, **AK1.Cu** = 20 μM, **JF-2** = 10 μM (840 nm), **JR-2** = 5 μM (840 nm), **JR-3** = 10 μM (840 nm), **JW-1** = 20 μM, **IG-1** = 20 μM, **FM4-64** = 20 μM (multimodal imaging), **FM4-64** = 10 μM (for comparison with di-4-ANEPPS, 840 nm), **di-4-ANEPPS** = 10 μM (840 nm), **RH123** = 20 μM, LysoTracker™ Yellow HCK-123 = 3 μM, and the ER-Tracker™ Red = 5 μM.

3. Supplemental synthetic procedures

General synthetic procedure: All commercial reagents and solvents were procured from Sigma Aldrich unless specified. The chloroform, dimethylformamide, pyridine, tetrahydrofuran, and dimethylsulfoxide were procured from Fisher Scientific, and dichloromethane was procured from Honeywell Riedel-de-Haën. Deuterated solvents were procured from Aldrich. The SX-1 resins for size-exclusion chromatography was procured from Bio-Beads® and the Dowex® chloride anion exchange resin were procured from Sigma Aldrich. The Geduran® Si 60 silica gel was used for flash column chromatography. Benchtop centrifuge from Eppendorf was used to wash the final compound **AK-1** with solvents during its purification. Compounds **1**, **2**, **JR-2** and **JR-3** were synthesized as per our previously reported literature procedure (Lopez-Duarte et al., 2013; Reeve et al., 2009).

Chemical reactions were performed under inert atmosphere (Ar gas) unless otherwise stated. NMR spectra were acquired on 400 MHz (Bruker AVIIIHD 400) and 500 MHz (Bruker AVII 500, Bruker AVIIIHD 500) spectrometers. Chemical shifts are reported in ppm relative to tetramethylsilane (TMS) as internal standard. MALDI-ToF (Waters MALDI micro) spectrometer was used for mass analysis.

3.1 Synthesis of JF-2 and JF-3

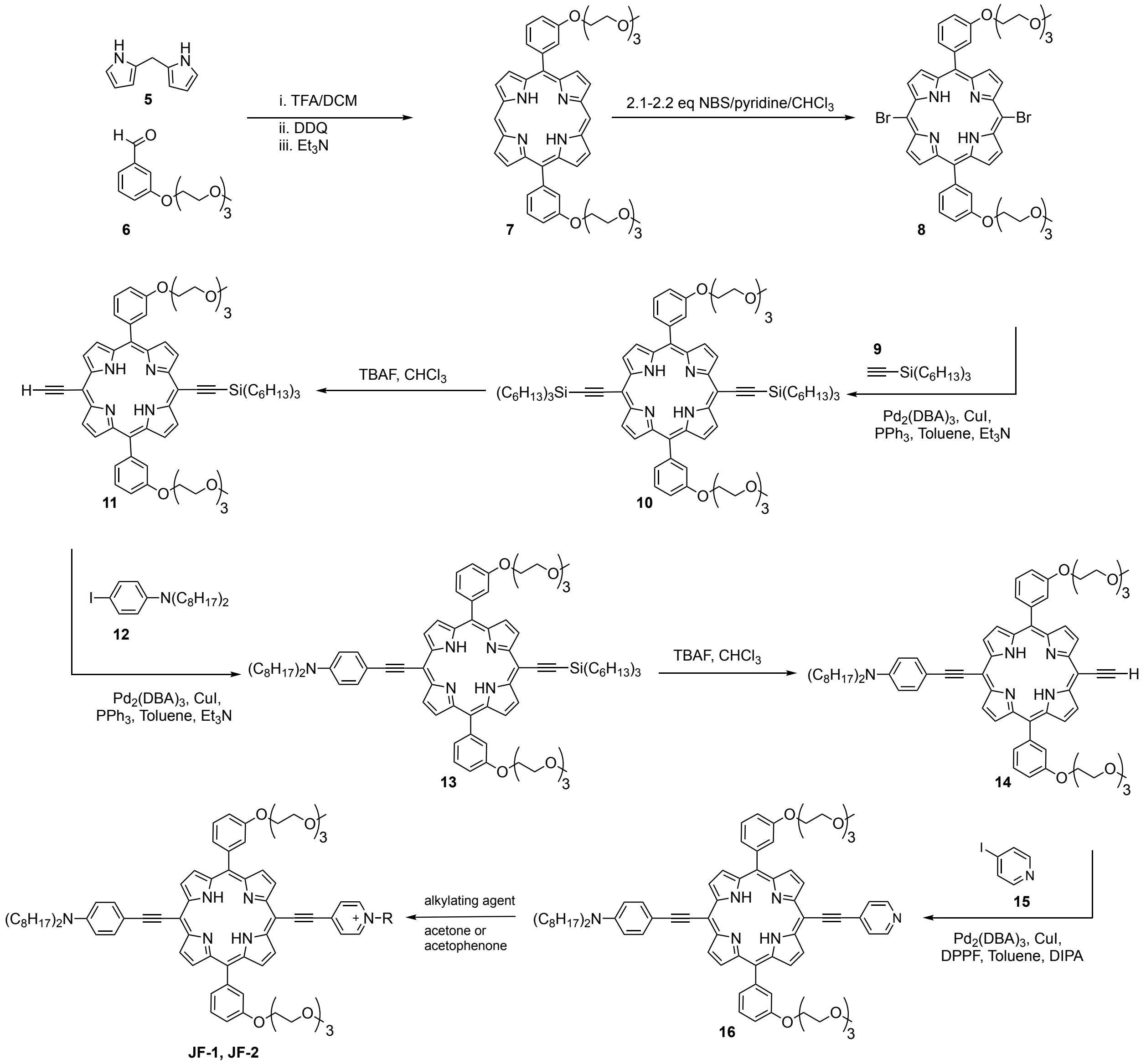

Scheme S1. Synthetic procedure for **JF-1** and **JF-2,** related to Figure 1. In the last step, 1-iodo-5-triethylammonium-pentane was used as the alkylating agent to synthesize **JF-1**, while 1,4-butane sultone was used to synthesize **JF-2**.

Compound **12** was synthesized according to the literature procedure (Tykwinski et al., 1996).

Compound 5: Dipyrromethane was synthesized as per literature procedure (Littler et al., 1999). Briefly, formaldehyde (33% w/w solution in water, 10.8 mL, 120 mmol) was added to pyrrole (200 mL, 2.88 mol) and the solution degassed by repeated evacuation and stirring under Ar at RT. Trifluoroacetic acid (1.08 mL, 14.1 mmol) was added by syringe under vigorous stirring and in the Ar atmosphere. The reaction proceeded for 5 min before CH_2Cl_2 (200 mL) was added, followed immediately by Na_2CO_3 (aq., sat., 200 mL). The organic layer was washed with Na_2CO_3 (aq.) (sat., 2 × 200 mL) and water (2 × 200 mL), then dried over Na_2SO_4. The solvent and then excess pyrrole were evaporated under reduced pressure. Distillation of the oily residue in a Kugelrohr apparatus (180 °C, 0.6 mbar) yielded the product **5** as a white solid. The product solidifies in the collecting vial into a robust stone difficult to remove. The convenient way to collect it is by washing with CH_2Cl_2. **Yield:** 6.9 g, 40%. **^{1}H NMR** (400 MHz, $CDCl_3$) δ/ppm: 7.76 (br s, 2 H, N*H*), 6.64 (m, 2 H, pyrrole **α**-*H*), 6.16 (m, 2 H, pyrrole **β**-*H*), 6.04 (m, 2 H, pyrrole **β**-*H*), 3.96 (s, 2 H, C*H₂*). **^{13}C NMR** (100 MHz, $CDCl_3$) δ/ppm: 121.2, 117.4, 108.4, 106.5, 26.4.

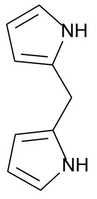

Porphyrin 7: This compound was prepared by adapting a literature procedure (Balaz et al., 2009). Dipyrromethane **5** (2.34 g, 16.0 mmol) and 3-(2-[2-(2-methoxy-ethoxy)-ethoxy]-ethoxy)-benzaldehyde **6** (4.30 g, 16.0 mmol) were dissolved in DCM (2.4 L). The solution was stirred vigorously and degassed by bubbling with N_2 for 0.5 h and trifluoroacetic acid (1.2 mL) was added *via* syringe under gentle bubbling of N_2. The flask was shielded from light with and the solution stirred at room temperature for 3.5 h. 2,3-Dichloro-5,6-dicyano-1,4-benzoquinone (DDQ, 4.40 g, 19.4 mmol) was added and the solution stirred for a further 30 min. The mixture was neutralized with triethylamine (25 mL), the crude mixture was concentrated to 500 mL and then poured directly onto a silica gel pad (50 cm × 4 cm) packed in DCM. Fast-running DDQ residues were removed with DCM and the product eluted with 99:1 DCM:MeOH. A second flash chromatography of increasing polarity (SiO_2; DCM:EtOAc 9:1 to 5:1 to 3:1) was performed to ensure that all tarry residues and side products were removed. On removal of the solvent and drying under high vacuum, product **7** was obtained as a purple solid glass. **Yield:** 2.0 g, 30 %. **^{1}H NMR** (500 MHz, $CDCl_3$/1% pyridine) δ/ppm: –3.09 (br. s, 2H, NH), 3.30–3.35 (m, 6H, OCH_3), 3.46–3.52 (m, 4H, OCH_2), 3.60–3.65 (m, 4H, OCH_2), 3.66–3.71 (m, 4H, OCH_2), 3.74–3.80 (m, 4H, OCH_2), 3.89–3.97 (m, 4H, OCH_2), 4.30–4.37 (m, 4H, OCH_2), 7.36–7.41 (m, 2H, CH), 7.66–7.72 (m, 2H, CH), 7.85–7.90 (m, 4H, CH), 9.12–9.15 (m, 4H, CH), 9.37 (d, *J* = 4.5 Hz, 4H, CH), 10.27–10.31 (m, 2H, CH). **^{13}C NMR** (125 MHz, $CDCl_3$/ 1% pyridine-d_5) δ/ppm: 59.0 (OCH_3), 67.7, 69.8, 70.5, 70.6, 70.9, 71.9 (OCH_2), 105.3, 114.2, 118.8, 121.4, 127.7, 128.0, 131.1, 131.6, 142.6, 145.2, 147.0, 157.5 (CH_{Ar}, C_{Ar}). ***m/z* (MALDI-TOF):** 786.59 ($C_{46}H_{50}N_4O_8$, $[M]^+$, requires 786.36, 100%); ***m/z* (HRMS, MICRO-TOF):** 809.3522 ($C_{46}H_{50}N_4NaO_8$, $[M+Na]^+$, requires 809.3521).

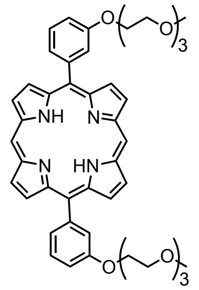

Porphyrin 8: This compound was prepared by adapting literature procedure (Balaz et al., 2009). Porphyrin **7** (1.0 g, 1.27 mmol) was dissolved in chloroform (100 mL) with pyridine (0.7 mL). A solution of NBS (2.1 eq., 480 mg, 2.7 mmol) in chloroform (50 mL) and pyridine (0.4 mL) was added dropwise over 60 min. The mixture was stirred for 1 h and the progress was monitored by TLC (SiO_2; DCM:EtOAc 3:1 or DCM:acetone 20:1). After quenching with acetone (5 mL), the solvents were removed under reduced pressure; the crude material was dissolved in toluene and extracted three to four times with water to remove the *N*-hydroxysuccinimide. After the product **8** was eluted from a silica column with (SiO_2; DCM: EtOAc 3:1) and evaporation of

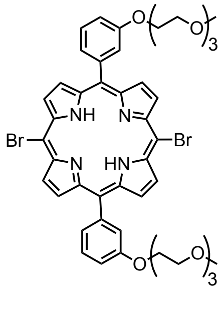

the solvent, porphyrin **8** was obtained in form of a purple viscous oil. **Yield:** 1.21 g, 95–99 %. ^{1}**H NMR** (500 MHz, $CDCl_3$/ 1% pyridine-*d*$_5$) δ/ppm: –2.76 (br. s, 2H), 3.32 (s, 6H), 3.47–3.51 (m, 4H), 3.60–3.64 (m, 4H), 3.67–3.71 (m, 4H), 3.76–3.80 (m, 4H), 3.93–3.98 (m, 4H), 4.30–4.39 (m, 4H), 7.35–7.41 (m, 2H), 7.61–7.69 (m, 2H), 7.72–7.78 (m, 4H), 8.82–8.95 (m, 4H, *J* = 4.4 Hz), 9.59 (d, 4H, *J* = 4.8 Hz). ^{13}**C NMR** (125 MHz, $CDCl_3$/ 1% pyridine-*d*$_5$) δ/ppm: 59.0 (OCH_3), 67.7, 69.8, 70.5, 70.6, 70.9, 71.9 (OCH_2), 103.7, 114.4, 121.0, 121.2, 127.6, 127.7, 132.4, 142.5, 157.2 (CH_{Ar}, C_{Ar}). ***m/z*** **(MALDI-TOF):** 944.95 ($C_{46}H_{48}Br_2N_4O_8$, $[M]^+$, requires 944.18, 100%); ***m/z*** **(HRMS, MICRO-TOF):** 965.1728 ($C_{46}H_{48}Br_2N_4NaO_8$, $[M+Na]^+$, requires 965.1731).

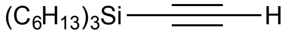

Compound 9: Chlorotrihexylsilane (15.2 mL, 41.6 mmol) was added dropwise under Ar to a stirred solution of ethynylmagnesium bromide (0. 50 M in THF, 100 mL, 50.0 mmol). The reaction mixture was heated at reflux for 1 h before HCl (aq.) (10%, 80 mL) was added. The organic layer was washed with water (80 mL) and dried over Na_2SO_4. The product was dried at a reduced pressure of 0.4 mbar for 30 min to yield a yellow oil. **Yield**: 10.0 g, 77.9%. ^{1}**H NMR** (400 MHz, $CDCl_3$) δ/ppm: 2.35 (s, 1 H, ≡C*H*), 1.41–1.22 (m, 24 H, C*H$_2$*), 0.89–0.83 (m, 9 H, C*H*), 0.63–0.56 (m, 6 H, C*H*). ^{13}**C NMR** (100 MHz, $CDCl_3$) δ/ppm: 94.1, 88.5, 33.3, 31.6, 23.9, 22.7, 14.3, 13.2. ***m/z*** **(ESI$^-$)** 307.3 ($C_{20}H_{40}Si$, M requires 308.3).

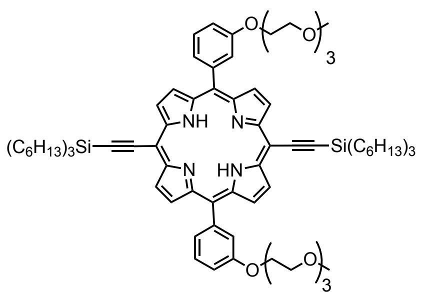

Porphyrin 10: The dibrominated porphyrin **8** (0.99 g, 1.05 mmol), trihexylsilylacetylene **9** (1.0 g, 3.14 mmol), $Pd_2(dba)_3$ (30 mg, 0.03 mmol), triphenylphosphine (60 mg, 0.21 mmol) and copper(I) iodide (20 mg, 0.1 mmol) were dried under vacuum in a Schlenk tube and flushed with argon. Toluene (20 mL) and triethylamine (10 mL) were added by syringe and solution was degassed by three freeze-thaw cycles. Once it had returned to room temperature, the mixture was stirred for 0.5 h and then heated to 40 °C until no further change was observed (ca. 3 h; TLC: DCM:EtOAc; 15:1). After the reaction mixture was cooled to room temperature, it was diluted with toluene (100 mL) and added into a separating funnel that was filled with 150 mL saturated ammonium chloride solution. The mixture was washed several times with water and the solvent was evaporated. A silica column with 15:1 DCM:EtOAc as the eluent gave the porphyrin **10** as a purple viscous oil. **Yield:** 1.32 g, 89%. ^{1}**H NMR** (500 MHz, $CDCl_3$ / 1% pyridine-*d*$_5$) δ/ppm: –2.12 (br. s, 2H, NH), 0.90–0.98 (m, 18H, CH_3), 1.03–1.09 (m, 12H, CH_2), 1.35–1.49 (m, 24H, CH_2), 1.55–1.63 (m, 12H, CH_2), 1.76–1.85 (m, 12H, CH_2), 3.34 (s, 6H, OCH_3), 3.49–3.53 (m, 4H, OCH_2), 3.62–3.67 (m, 4H, OCH_2), 3.69–3.75 (m, 4H, OCH_2), 3.79–3.84 (m, 4H, OCH_2), 3.96–4.02 (m, 4H, OCH_2), 4.34–4.40 (m, 4H, OCH_2), 7.37–7.43 (m, 2H, CH), 7.69 (t, 4H, *J* = 7.6 Hz, CH), 7.79–7.83 (m, 4H, CH), 8.90 (d, 4H, *J* = 4.6 Hz, CH), 9.66 (d, 4H, *J* = 4.7 Hz, CH). ^{13}**C NMR** (125 MHz, $CDCl_3$/ 1% pyridine-*d*$_5$) δ/ppm: 13.8, 14.2, 22.7, 24.4, 31.7, 33.3 (CH_3, CH_2), 59.0 (OCH_3), 67.8, 69.9, 70.5, 70.7, 70.9, 71.9 (OCH_2), 101.1, 101.4, 107.9, 114.4, 121.1, 121.4, 127.67, 127.69, 130.7 br., 131.6 br., 142.6, 157.4 (C≡C, CH_{Ar}, C_{Ar}). ***m/z*** **(MALDI-TOF)** 1401.54 ($C_{86}H_{127}N_4O_8Si_2$, [M+H]+, requires 1400.92, 100%); ***m/z*** **(HRMS, MICRO-TOF):** 1421.8981 ($C_{86}H_{126}N_4NaO_8Si_2$, $[M+Na]^+$, requires 1421.9006).

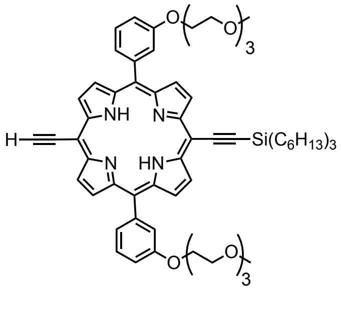

Mono desilylated porphyrin 11: Porphyrin **7** (0.44 g, 0.314 mmol), was dissolved in chloroform (100 mL) and a solution of 0.6 eq. of TBAF (0.2 mL; 1.0 M in THF) was added slowly. The progress was monitored by TLC (SiO_2: DCM:EtOAc; 10:1; 3:1). Once the mixture showed first indications of

the double deprotected derivative the mixture was quenched with acetic acid (12 µL, 0.2 mmol). After 5 min, MeOH (10 mL) was added and the mixture was then passed through a short column of SiO_2. The solvent was evaporated under reduced pressure and the remaining crude mixture was purified by flash chromatography on silica, eluting with DCM:EtOAc of increasing polarity (15:1 → 10:1). Hereby the unreacted starting material (**7**, 241 mg, 55% yield) eluted as first, followed from the mono (**11**, 100 mg, 28% yield) and double deprotected porphyrin. The alkyne **11** has limited stability under normal laboratory conditions, so it is normally prepared and used immediately (without further purification and characterization) in the following coupling step, for this reason the product is dried in a Schlenk tube, ready for use in the next step. It can be stored overnight as a dry solid at –20 °C.

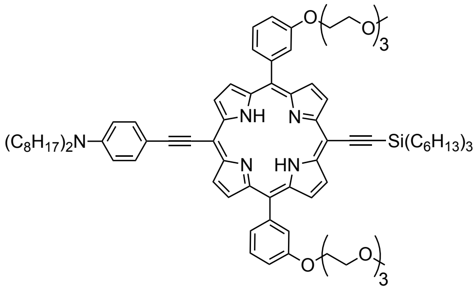

Donor substituted porphyrin 13: The mono-deprotected porphyrin **11** (200 mg, 0.18 mmol), 1-iodo-4-*N*,*N*-dioctylamino-benzene **12** (160 mg, 0.36 mmol), $Pd_2(dba)_3$ (4.1 mg, 9 µmol), triphenylphosphine (10 mg, 36 µmol) and copper(I) iodide (4.0 mg, 18 µmol) were dried under vacuum in a Schlenk tube and flushed with argon. Toluene (7 mL) and triethylamine (4 mL) were added by syringe and solution was degassed by three freeze-thaw cycles. Once it had returned to room temperature, the mixture was stirred at for 0.5 h and then heated to 40 °C until no further change was observed (2–3 h; TLC: DCM:EtOAc; 20:1). After the reaction mixture was cooled to room temperature, it was diluted with toluene (100 mL) and added into a separating funnel that was filled with 150 mL saturated ammonium chloride solution. The mixture was washed several times with water and the solvent was evaporated. A silica column with 20:1 DCM: EtOAc as the eluent gave the porphyrin **13** as a green glass. **Yield**: 208 mg, 81%. **^{1}H NMR** (400 MHz, $CDCl_3$ / 1% pyridine-d_5) δ/ppm: –1.89 (br. s, 2H, NH); 0.87–0.94 (m, 15H; CH_3), 0.99–1.05 (m, 6H; CH_2), 1.25–1.44 (m, 32H; CH_2), 1.51–1.59 (m, 6H; CH_2), 1.64–1.72 (m, 4H; CH_2), 1.72–1.80 (m, 6H; CH_2), 3.31(s, 6H; OCH_3), 3.35–3.41 (m, 4H; NCH_2), 3.47–3.51 (m, 4H, OCH_2), 3.61–3.65 (m, 4H, OCH_2), 3.68–3.72 (m, 4H, OCH_2), 3.77–3.81 (m, 4H, OCH_2), 3.95–3.99 (m, 4H, OCH_2), 4.32–4.37 (m, 4H, OCH_2), 6.79 (d, 2H, *J* = 8.5 Hz, CH), 7.35–7.39 (m, 2H, CH), 7.63–7.67 (m, 2H, CH), 7.75–7.79 (m, 4H, CH), 7.86 (d, 2H, *J* = 8.5 Hz, CH), 8.82 (d, 4H, *J* = 4.6 Hz, CH), 9.56 (d, 2H, *J* = 4.7 Hz, CH), 9.66 (d, 2H, *J* = 4.6 Hz, CH). **^{13}C NMR** (125 MHz, $CDCl_3$/ 1% pyridine-d_5) δ/ppm: 13.8, 14.1, 14.2, 22.7, 24.3, 27.2, 27.3, 29.3, 29.5, 31.6, 31.8, 33.3 (CH_3, CH_2), 51.1 (NCH_2), 59.0 (OCH_3), 67.7, 69.9, 70.5, 70.6, 70.9, 71.9 (OCH_2), 90.2, 100.0, 100.8, 103.7, 108.0, 109.1, 111.5, 114.4, 121.0, 121.2, 127.61, 127.63, 133.1, 142.7, 148.4, 157.3 (C≡C, CH_{Ar}, C_{Ar}). ***m/z* (MALDI-TOF):** 1432.70 ($C_{90}H_{125}N_5O_8Si_2$, [M]+, requires 1432.93, 100%); ***m/z* (HRMS, MICRO-TOF)**: 1454.9175 ($C_{90}H_{125}N_5NaO_8Si_2$, [M+Na]+, requires 1454.9190).

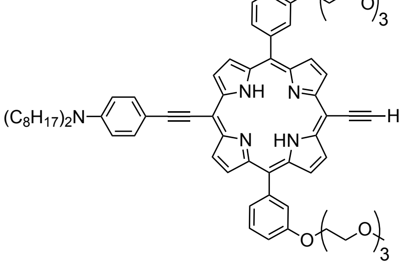

Desilylated porphyrin 14: The porphyrin **13** (160 mg, 0.11 mmol), was dissolved in chloroform (50 mL) and degassed by gentle bubbling with nitrogen for 10 min. To this solution 2 eq. of a solution of TBAF (0.22 mL, 1.0 M in THF) was added slowly and the progress was monitored by TLC (SiO_2: DCM: EtOAc; 20:1; 10:1). Once the mixture was completely desilylated it was quenched by equimolar amounts of glacial acid and stirred for 5 min MeOH (5 mL) was added and the mixture was then plugged over SiO_2. Compound **14** has similar to **11** limited stability under normal laboratory conditions, so it is normally prepared and used immediately (without further purification and characterization) in the following

coupling step, for this reason the product is dried in a Schlenk tube, ready for use in the next step. Due to a TLC clean cleavage reaction, the theoretical yield was assumed to be 100% (127 mg). It can be stored overnight as a dry solid at –20 °C.

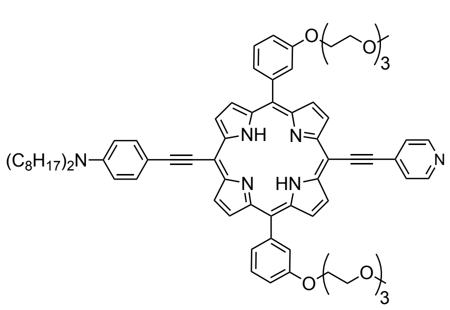

Donor acceptor substituted porphyrin 16: The desilylated porphyrin **14** (127 mg, 0.11 mmol), 1-iodo-pyidine **15** (120 mg, 0.58 mmol), $Pd_2(dba)_3$ (2.7 mg, 3 µmol), bis-diphenylphosphino-ferrocene (DPPF) (3.5 mg, 1.5 µmol) and copper(I) iodide (2.2 mg, 6 µmol) were dried under vacuum in a Schlenk tube and flushed with argon. Toluene (7 mL) and diisopropylamine (4 mL) were added by syringe and solution was degassed by three freeze-thaw cycles. Once it had returned to room temperature, the mixture was stirred at for 0.5 h and then heated to 40 °C until no further change was observed (1–2 h; TLC: DCM:MeOH; 20:1). After the reaction mixture was cooled to room temperature, it was diluted with toluene (100 mL) and added into a separating funnel that was filled with 150 mL saturated ammonium chloride solution. The mixture was washed several times with water and the solvent was evaporated. A subsequent chromatography on silica (20:1 chloroform:MeOH); BIO-Beads® S-X1 (size-exclusion; 200–400 mesh, toluene:pyridine; 100:1) and silica (30:1 chloroform:MeOH) gave **16** as a green glass. **Yield:** 115 mg, 85%. ^{1}**H NMR** (500 MHz, $CDCl_3$ / 1% pyridine-d_5) δ/ppm: –1.81 (br. s, 2H, NH); 0.87–0.94 (m, 6H; CH_3), 1.21–1.42 (m, 20H; CH_2), 1.62–1.72 (m, 4H; CH_2), 3.30 (s, 6H; OCH_3), 3.34–3.40 (m, 4H; NCH_2), 3.45–3.50 (m, 4H, OCH_2), 3.59–3.64 (m, 4H, OCH_2), 3.67–3.71 (m, 4H, OCH_2), 3.76–3.80 (m, 4H, OCH_2), 3.93–3.98 (m, 4H, OCH_2), 4.32–4.37 (m, 4H, OCH_2), 6.77 (d, 2H, J = 8.6 Hz, CH), 7.34–7.39 (m, 2H, CH), 7.62–7.76 (m, 2H, CH), 7.74–7.78 (m, 4H, CH), 7.80–7.86 (m, 4H, CH), 8.76–8.82 (m, 4H, CH), 8.85 (d, 2H, J = 4.5 Hz, CH), 9.55 (d, 2H, J = 4.5 Hz, CH), 9.64 (d, 2H, J = 4.5 Hz, CH). ^{13}**C NMR** (125 MHz, $CDCl_3$/ 1% pyridine-d_5) δ/ppm: 14.1(CH_3), 22.6, 27.1, 27.3, 29.3, 29.5, 31.8 (CH_2), 51.0 (NCH_2), 58.9 (OCH_3), 67.7, 69.8, 70.5, 70.6, 70.9, 71.8 (OCH_2), 90.3, 93.8, 96.8, 97.7, 100.7, 104.7, 108.8, 111.5, 114.4, 121.0, 121.7, 125.3, 127.6, 132.0, 133.2, 142.5, 148.5, 150.0, 157.3 (C≡C, CH_{Ar}, C_{Ar}). *m/z* (MALDI-TOF) 1228.27 ($C_{77}H_{91}N_6O_8$, $[M+H]^+$, requires 1228.7, 100%); ***m/z* (HRMS, MICRO-TOF):** 1249.6880 ($C_{77}H_{90}N_6NaO_8$, $[M+Na]^+$, requires 1249.6712);

General procedure for the alkylation with 1-iodo-5-triethylammonium-pentane:

The doubly charged compounds **JF-1** and **JF-1.Cu** were prepared by mixing precursors **16, 16.Cu** (approx. 50 mg) with and an excess of 1-iodo-5-triethylammonium-pentane (approx. 500 mg) in 2-pentanone (4 mL). The mixture was heated under argon to 80–90 °C. The reaction progress was monitored by TLC (SiO_2: chloroform:MeOH; 20:1) and after the most of the starting material was consumed (approx. 4 h), the solvent was removed under reduced pressure. Washing the crude mixture of **JF-1.Cu** on a filter paper with water allowed removing the excess of 1-iodo-5-triethylammonium-pentane, whereas the porphyrin free base **JF-1** was dissolved. The remaining metallic green (**JF-1, JF-1.Cu**) crude mixture was dissolved in NH_4Cl saturated water-methanol mixture (90:1) and extracted using chloroform/ethanol mixtures until the aqueous layer was mostly decolored. The solvents were removed under reduced pressure and the crude mixture was redissolved in NH_4Cl saturated water-methanol mixture (90:1) and extracted with chloroform/ethanol. After evaporation of the solvent, the reaction mixture was dissolved in toluene and filtered from the ammonium chloride. A further purification by DOWEX 50 ion exchange resin (MeOH) and BIO-Beads® SX-1 size-exclusion (200–400 mesh) using toluene as solvent, microfiltration and precipitation from toluene using *n*-hexane as antisolvent yielded the doubly charged compounds. The analytical purity was determined by NMR.

5-Iodo-Triethylammonium-pentane-iodide: The compound was prepared adapting a literature procedure for similar compounds (Sebastiano et al., 2001). A solution of acetone (100 mL), 1,5-diiodopentane (16.2 g, 0.05 mol, 7.44 mL) and triethylamine (5.06 g, 0.05 mol, 7.0 mL) was vigorously stirred at 20 °C for 24 h. The amount of the solvent was reduced to 25 mL and the solution was filtered from the precipitate. Addition of diethylether to the mother liquor precipitated 5-iodo-triethylammonium-pentane-iodide as pale yellow solid. **Yield:** 3.2 g, 15%. ^{1}**H NMR** (400 MHz, $CDCl_3$) δ/ppm: 1.39 (t, 9H, J = 6.2 Hz, NCH_3), 1.55 (quint, 2H, J = 7.2 Hz, CH_2), 1.75–1.87 (m, 2H, CH_2), 1.93 (quint, 2H, J = 7.1 Hz, CH_2), 3.22–3.28 (m, 2H, ICH_2), 3.30–3.37 (m, 2H, NCH_2), 3.50 (quint, 6H, J = 7.2 Hz, CH_2). ^{13}**C NMR** (120 MHz, DMSO-d_6) δ/ppm: 6.9 (ICH_2), 8.4 (CH_3), 21.3, 27.3, 32.3 (CH_2), 53.8, 57.6 (NCH_2); ***m/z*** **(HRMS, MICRO-TOF)**: 298.1018 ($C_{11}H_{25}$ IN, [M]+, requires 298.1026).

Double charged porphyrin free base JF-1: The reaction of porphyrin **16** (57 mg, 0.046 mmol) with 1-iodo-5-triethylammonium-pentane (500 mg, 1.2 mmol) in 2-pentanone (4 mL) yielded **JF-1**. **Yield:** 48 mg, 75%. ^{1}**H NMR** (500 MHz, DMSO-d_6) δ/ppm: –1.60 (br. s, 2H, NH); 0.86–0.93 (m, 6H; CH_3), 1.17–1.23 (m, 9H; CH_3), 1.25–1.43 (m, 22H; CH_2), 1.56–1.66 (m, 4H; CH_2), 1.66–1.75 (m, 2H, CH_2), 2.05–2.14 (m, 2H, CH_2), 3.12–3.21 (m, 8H; NCH_2, OCH_3), 3.26 (q, 6H, J = 7.2 Hz, NCH_2), 3.35–3.44 (m, 8H; NCH_2, OCH_2), 3.48–3.52 (m, 4H, OCH_2), 3.55–3.58 (m, 4H, OCH_2), 3.63–3.67 (m, 4H, OCH_2), 3.83–3.90 (m, 4H, OCH_2), 4.32–4.41 (m, 4H, OCH_2), 4.72 (t, 2H, J = 6.8 Hz, NCH_2), 6.87 (d, 2H, J = 8.7 Hz, CH), 7.48–7.53 (m, 2H, CH), 7.76–7.84 (m, 6H, CH), 7.92 (d, 2H, J = 8.7 Hz, CH), 8.82 (d, 2H, J = 4.5 Hz, CH), 8.88–8.93 (m, 4H, CH), 9.30 (d, 2H, J = 6.3 Hz, CH), 9.71 (d, 2H, J = 4.5 Hz, CH), 9.83 (d, 2H, J = 4.5 Hz, CH). ^{13}**C NMR** (125 MHz, DMSO-d_6) δ/ppm: 7.2, 14.0 (CH_3), 20.5, 22.1, 22.3, 26.4, 26.8, 28.7, 28.9, 30.1, 31.2 (CH_2), 50.1, 52.0, 55.7 (NCH_2), 58.0 (OCH_3), 60.1 (NCH_2), 67.6, 69.1, 69.6, 69.8, 70.0, 71.2 (OCH_2), 90.2, 93.4, 94.6, 102.9, 105.5, 106.1, 107.0, 111.6, 114.9, 120.7, 122.8, 127.2, 128.3, 129.1, 133.5, 139.1, 141.4, 144.6, 148.8, 157.2 (C≡C, CH_{Ar}, C_{Ar}). ***m/z*** **(MALDI-TOF):** 1431.53 ($C_{88}H_{114}ClN_7O_8$, $[M-HCl]^+$, requires 1432.84, 100%). ***m/z*** **(HRMS, MICRO-TOF):** 698.9380 ($C_{88}H_{115}N_7O_8$, $[M]^{2+}$, requires 698.9398). **UV-Vis** (DMF, 25 °C) λ_{max} (log **ε**): 448 (5.03); 642 (4.54); 727 (4.67).

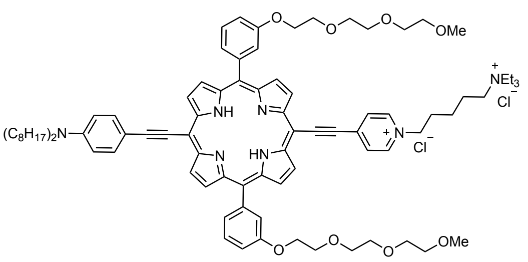

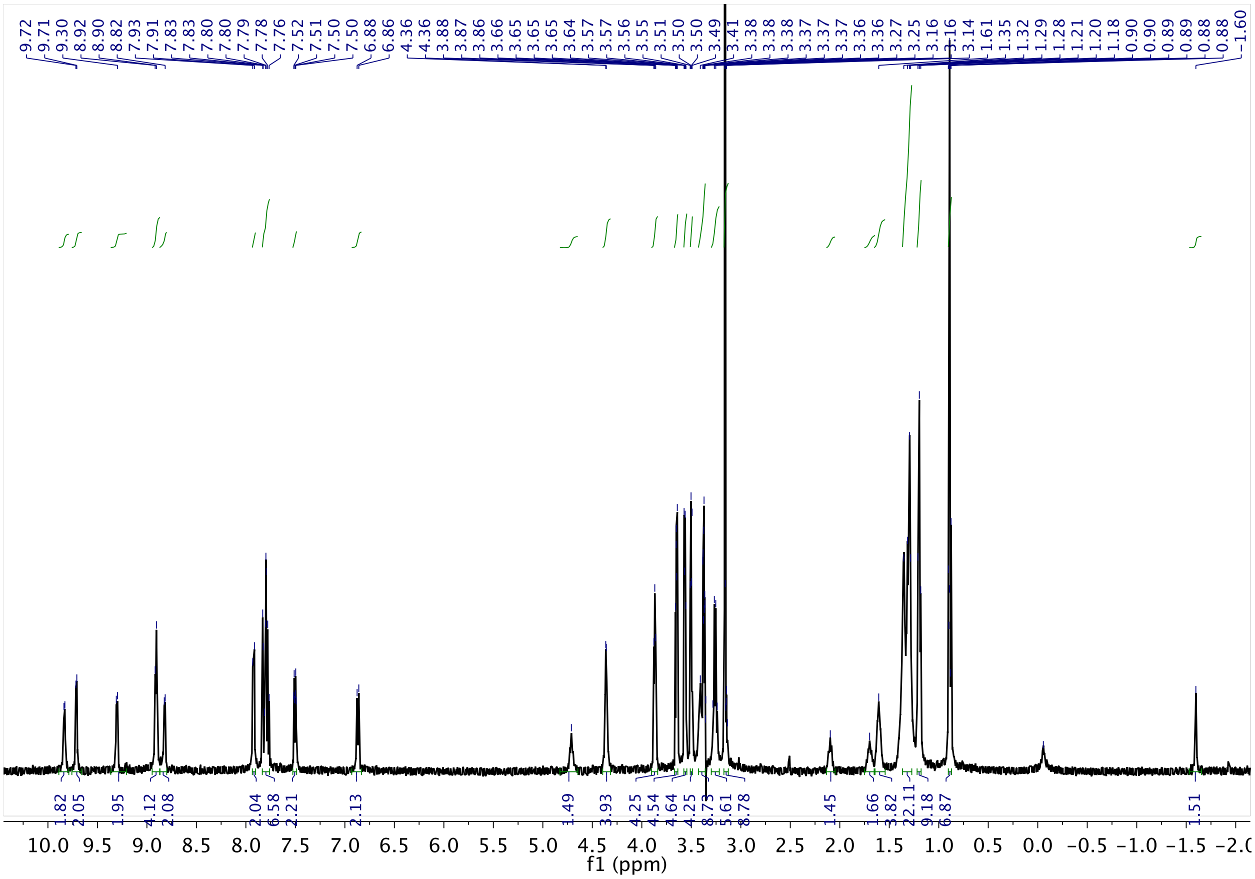

Figure S9. ^{1}H-NMR spectrum of **JF-1** (d_6-DMSO, 500 MHz), related to Figure 1.

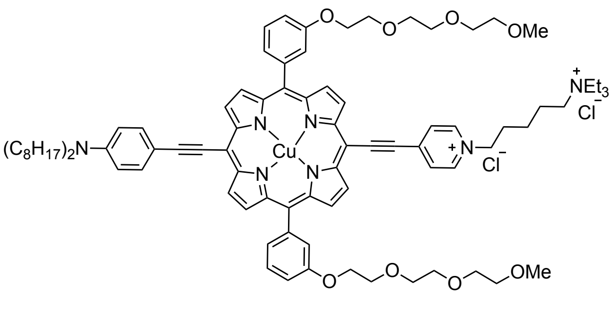

Double charged copper porphyrin JF-1.Cu: The reaction on octyl version of **16.Cu** (66 mg, 0.051 mmol) (synthesized by inserting copper in **16**) with 1-iodo-5-triethylammonium-pentane (514 mg, 1.2 mmol) in 2-pentanone (4 mL) gave **JF-1.Cu. Yield:** 61 mg, 78%. ***m/z* (MALDI-TOF):** 1494.37 ($C_{88}H_{112}ClCuN_7O_8$, $[M-HCl]^+$, requires 1494.76, 100%); ***m/z* (HRMS, MICRO-TOF):** 729.3995 ($C_{88}H_{113}CuN_7O_8$, $[M]^{2+}$, requires 729.3968). **UV-Vis** (DMF, 25 °C) λ_{max} (log **ε**): 449 nm (4.97); 686 nm (4.65).

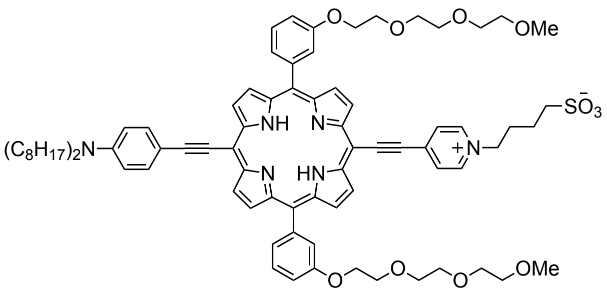

Porphyrin JF-2: To a solution of the corresponding porphyrin **16** (52 mg, 42.4 μmol) in acetophenone (2 mL) was added an excess of 1,4-butane sultone (1.2 mL, 11.7 mmol) and the resulting solution was vigorously stirred at 110–130 °C for approx. 5 h under Ar atmosphere with regular TLC monitoring (SiO_2: chloroform:MeOH; 20:1). After the starting material was almost consumed, the reaction was quenched by

evaporating the solvents. The slurry crude reaction mixture of **JF-2** was directly purified by BIO-Beads® S-X1 size-exclusion (200–400 mesh) using toluene as solvent. Microfiltration and precipitation of the evaporated prod from toluene using *n*-hexane as bad solvent yielded the charged compounds. **Yield:** 48 mg (83%). ^{1}**H NMR** (500 MHz, DMSO-d_6) δ/ppm: –1.85 (br. s, 2H, NH); 0.85–0.93 (m, 6H; CH_3), 1.21–1.37 (m, 20H; CH_2), 1.51–1.60 (m, 4H; CH_2), 1.68 (quint, 2H, J = 7.6 Hz, CH_2), 2.11 (quint, 2H, J = 7.6 Hz, CH_2), 2.55 (t, 2H, J = 7.6 Hz, SO_3CH_2), 3.16 (s, 6H; OCH_3), 3.30–3.39 (m, 8H; NCH_2, OCH_2), 3.48–3.52 (m, 4H, OCH_2), 3.55–3.59 (m, 4H, OCH_2), 3.63–3.67 (m, 4H, OCH_2), 3.85–3.89 (m, 4H, OCH_2), 4.32–4.40 (m, 4H, OCH_2), 4.64 (t, 2H, J = 6.8 Hz, NCH_2), 6.75 (d, 2H, J = 8.4 Hz, CH), 7.48–7.53 (m, 2H, CH), 7.69–7.84 (m, 8H, CH), 8.59 (d, 2H, J = 5.4 Hz, CH), 8.71–8.80 (m, 4H, CH), 9.19 (d, 2H, J = 6.4 Hz, CH), 9.49 (br s, 4H, CH). ^{13}**C NMR** (125 MHz, DMSO-d_6) δ/ppm: 14.0 (CH_3), 21.7, 22.1, 26.4, 26.8, 28.7, 28.9, 30.0, 31.3 (CH_2), 50.1, 50.4 (SO_3CH_2, NCH_2), 58.0 (OCH_3), 60.2 (NCH_2), 67.6, 69.1, 69.6, 69.8, 70.0, 71.2 (OCH_2), 90.0, 93.1, 94.4, 102.4, 105.1, 105.7, 107.0, 111.4, 114.8, 120.7, 122.6, 127.3, 128.2, 128.7, 133.3, 138.6, 141.4, 144.4, 148.6, 157.2 (C≡C, CH_{Ar}, C_{Ar}). ***m/z* (MALDI-TOF):** 1362.90 ($C_{81}H_{98}N_6O_{11}S$, [M]+, requires 1362.70, 100%); ***m/z* (HRMS, MICRO-TOF):** 1385.6862 ($C_{81}H_{98}N_6NaO_{11}S$, $[M+Na]^+$, requires 1385.6906). **UV-Vis** (DMF, 25 °C) λ_{max} (log **ε**): 446 (5.05); 640 (4.58); 726 (4.72).

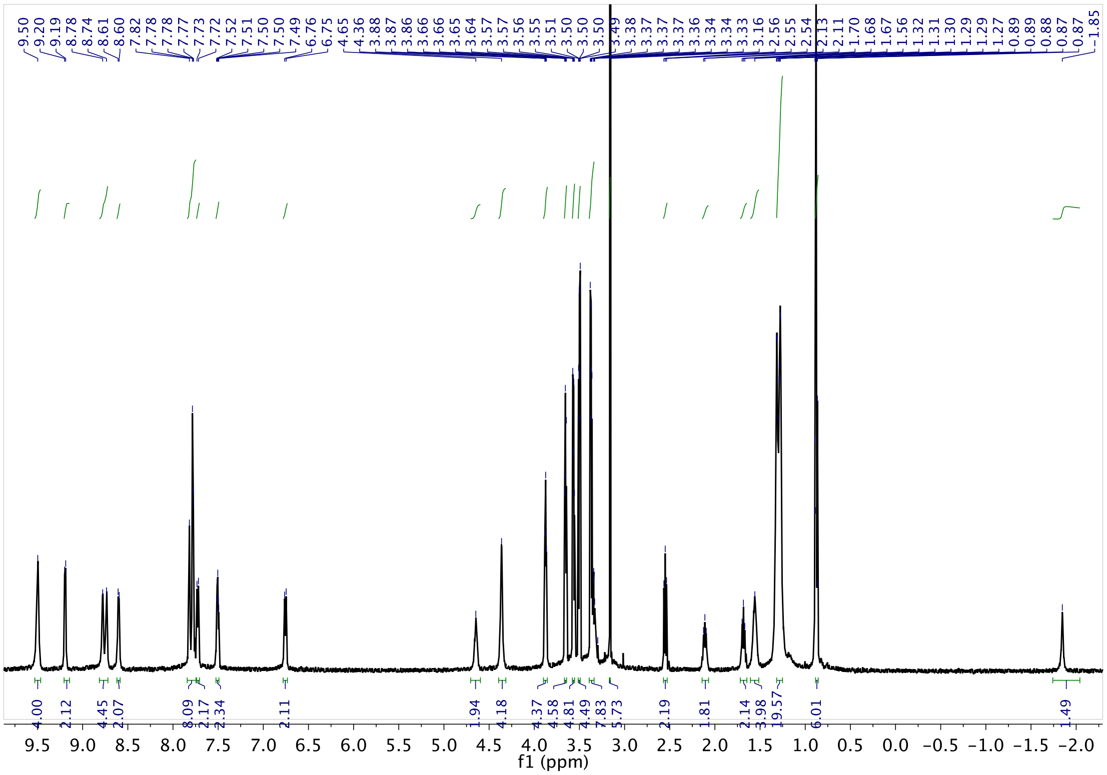

Figure S10. ^{1}H NMR spectrum of **JF-2** (d_6-DMSO, 500 MHz, DOSY experiment), related to Figure 1.

3.2 Synthesis of AK-1 and AK-1.Cu

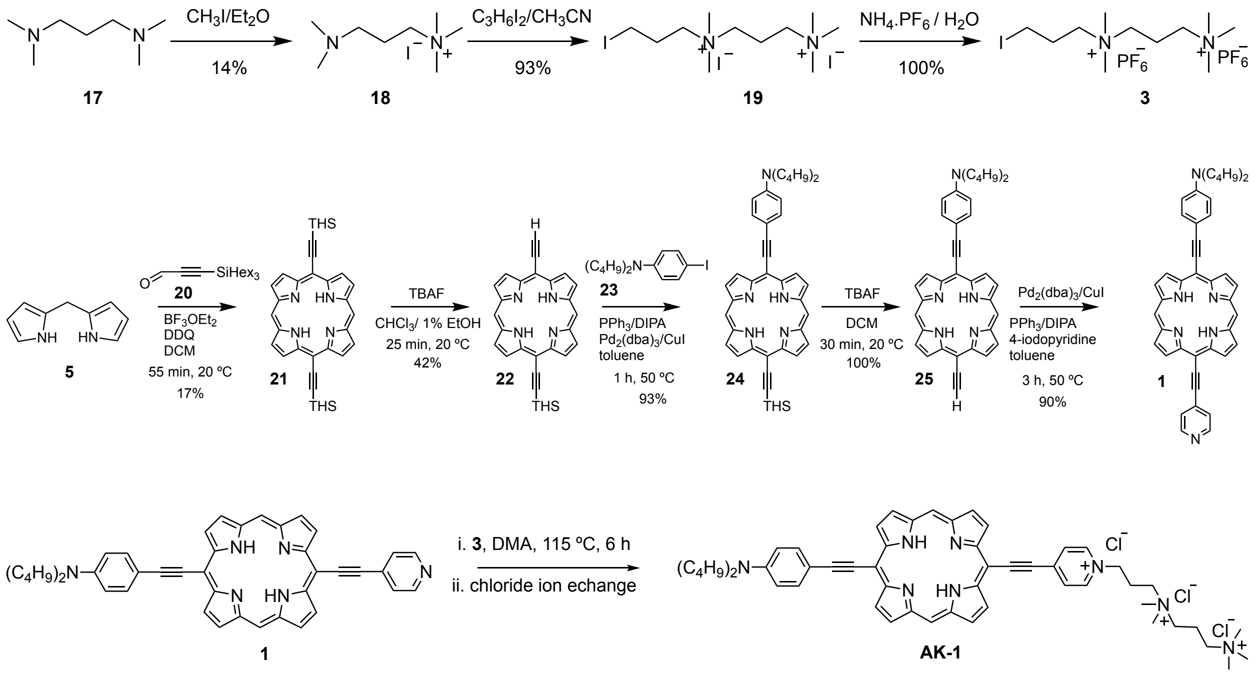

Scheme S2. Synthetic procedure for **AK-1**, Related to Figure 1.

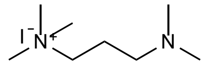

Compound 18: Compound **18** was synthesized according to the literature procedure (Yi et al., 2016). Briefly, *N*,*N*,*N'*,*N'*-tetramethyl-1,3-propanediamine **17** (5.0 g) was dissolved in diethyl ether (100 mL) and stirred. Methyl iodide (2.38 mL, 1 eq.) was added dropwise and the reaction mixture was stirred for 20 min until white precipitate formed. The white precipitate was washed with water (100 mL) three times and dried under high vacuum to yield **18** as a white amorphous powder. **Yield:** 1.45 g, 14%. ^{1}**H NMR** (400 MHz, D_2O) δ/ppm: 3.31 (m, 2H), 3.12 (s, 9H), 2.41 (m, 2H), 2.21 (s, 6H), 1.96 (m, 2H). ^{13}**C NMR** (100 MHz, D_2O) δ/ppm: 64.7, 54.6, 52.8, 43.6, 20.1. **m/z (ESI+)** 145.2, 146.2 ($C_8H_{21}N_2^+$ M^+ requires 145.2, $C_8H_{22}N_2^+$ $[M+H]^+$ requires 146.2).

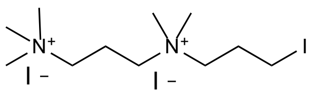

Compound 19: 3-(Dimethylamino)-*N*,*N*,*N*-trimethylpropan-1-aminium iodide **18** (600 mg, 2.2 mmol) was dissolved in acetonitrile (5 mL) and stirred followed by addition of 1,3-diiodpropane (2.7 mL, 22.0 mmol, 10 eq.). The reaction mixture was refluxed for 24 h. The solvent was evaporated under reduced pressure to form a yellow solid powder, which was washed with acetone to give **19** as a white powder. **Yield:** 1.2 g, 93%. ^{1}**H NMR** (400 MHz, D_2O) δ/ppm: 3.52 (m, 2H), 3.45 (m, 4H), 3.29 (t, 2H, ^{3}J = 6.4 Hz), 3.21 (s, 9H), 3.18 (s, 6H), 2.36 (m, 4H). ^{13}**C NMR** (100 MHz, D_2O) δ/ppm: 62.3, 64.9, 60.0, 53.2, 51.0, 25.5, 17.0, –0.3. ***m/z* (ESI+)** 441.0 ($C_{11}H_{27}I_2N_2^+$, M^+ requires 441.0).

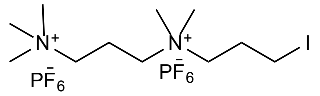

Compound 3: *N*^{1}-(3-Iodopropyl)-*N*^{1},*N*^{1},*N*^{3},*N*^{3},*N*^{3}-pentamethylpropane-1,3-bis(aminium)-diiodide **19** (1.0 g, 1.7 mmol) was dissolved in water just below saturation concentration. Ammonium hexafluorophosphate (900 mg, 5.1 mmol, 3 eq.) solution in water was added dropwise and stirred at RT for 15 min to form

precipitates. The precipitate was washed with water (100 mL) and dried under high vacuum to give product **3** as white solid. ^{1}**H NMR** (400 MHz, d_6-DMSO) δ/ppm: 3.38 (m, 2H), 3.31 (m, 4H), 3.24 (t, 2H, ^3J = 6.8 Hz), 3.11 (s, 9H), 3.09 (s, 6H), 2.22 (m, 4H). ^{13}**C NMR** (100 MHz, d_6–DMSO) δ/ppm: 63.9, 61.8, 59.6, 52.6, 50.6, 25.8, 16.8, 1.5. ***m/z* (ESI+)** 459.0, 460.0.0 ($C_{11}H_{27}F_6IPN_2^+$ M$^+$ requires 459.0, $C_{11}H_{28}F_6IPN_2^+$ [M+H]$^+$ requires 460.0).

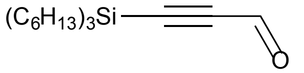

Compound 20: Compound 20 was synthesized as per literature procedure (Reeve et al., 2009). *n*-Butyl lithium (11.2 mL, 2.5 M solution in hexane) was added dropwise to a stirred solution of trihexylsilyl acetylene **9** (6.6 g, 21.3 mmol), in dry THF (18 mL) at 0 °C. The mixture was stirred for 15 min at 0 °C and then another 15 min at RT. The reaction mixture was transferred via cannula to a stirred solution of DMF (5 mL, mmol) in dry THF (18 mL) and stirred for 2 h at −80 °C. The reaction mixture was quenched with HCl (10% v/v, 50 mL), washed with H_2O and extracted with Et_2O. The solution was dried over Na_2SO_4 and concentrated to give **20** as yellow oil. **Yield:** 6.71 g, 93.5%. ^{1}**H NMR** (400 MHz, $CDCl_3$) δ/ppm: 9.17 (s, 1 H, C*H*O), 1.45–1.20 (m, 24 H, 2-5 hexyl-*H*), 0.89 (t, 9 H, ^3J = 6.7 Hz, 6 hexyl-*H*), 0.68 (m, 6 H, 1-hexyl-*H*). ^{13}**C NMR** (100 MHz, $CDCl_3$) δ/ppm: 175.9, 103.6, 102.5, 33.1, 31.5, 23.8, 22.7, 14.2, 12.6.

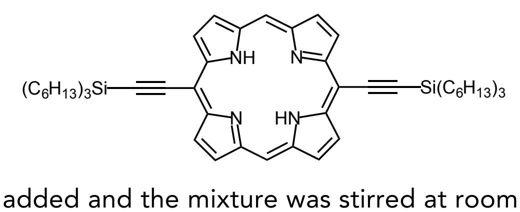

Porphyrin 21: Porphyrin **21** was prepared according to an adapted literature procedure (Anderson, 1992). Dipyrromethane (1.55 g, 10.60 mmol) was dried *in vacuo* for 1 h before addition of dry CH_2Cl_2 (600 mL) and trihexylsilyl propynal (3.7 g, 11.00 mmol). The solution was freeze-pump-thaw-degassed and $BF_3.OEt_2$ (450 µL, 3.64 mmol) was added and the mixture was stirred at room temperature for 45 min in the dark. After this time, DDQ (3.43 g, 15.11 mmol) was added and the mixture was stirred under air for 10 min. The crude mixture was passed through a large silica plug (CH_2Cl_2) and further purified by flash chromatography on silica (4:1 40–60 °C petrol ether: CH_2Cl_2). Fractions were evaporated to give **21** as a purple oil. **Yield:** 1.65 g, 16.8%. ^{1}**H NMR** (400 MHz, $CDCl_3$ with 1% C_5D_5N) δ/ppm: 10.09 (s, 2 H, meso-*H*), 9.67 (d, 4 H, ^3J = 4.5 Hz, **β**- *H*), 9.28 (d, 4 H, ^3J = 4.5 Hz, **β**- *H*), 1.86–1.74 (m, 12 H, hexyl-H), 1.64–1.54 (m, 12 H, hexyl-H), 1.50–1.35 (m, 24 H, hexyl-H), 1.10-1.02 (m, 12 H, hexyl-H), 0.93 (t, 18 H, ^3J = 7.06 Hz, hexyl-H).

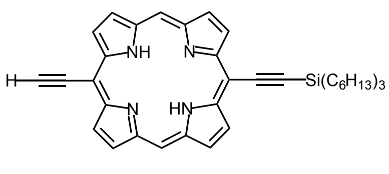

Porphyrin 22: Porphyrin **22** was prepared according to literature procedure (Reeve et al., 2009). Amylene stabilized $CHCl_3$ was passed through alumina and then mixed with 1% of dry EtOH. Porphyrin **21** (500 mg, 0.54 mmol) was dissolved in the $CHCl_3$ (25 mL). The solution was put under Ar before *n*-Bu_4NF (0.54 mL, 1 M in THF,) was added. The reaction was carefully monitored by TLC (PET ether 40- 60 °C : EtOAc 10 : 1) - spotted every 10 min. When starting material and monodeprotected product appeared roughly equal in intensity, the reaction was quenched by pouring directly onto a silica plug in CH_2Cl_2. Crude reaction mixture was purified by flash chromatography on SiO_2 (PET ether 40–60 °C : EtOAc 20 : 1 : 1). Fractions containing monodeprotected porphyrin **22** were evaporated to dryness to give a purple glass. **Yield:** 145 mg, 42%. ^{1}**H NMR** (400 MHz, $CDCl_3$) δ/ppm: 9.83 (s, 2H, meso-H), 9.57 (d, 2H, ^3J = 4.3 Hz, **β**-H), 9.52 (m, 2H, **β**-H), 9.13 (m, 4H, **β**-H), 4.21 (s, 1H, acetylene-H), 1.92–1.82 (m, 6H, hexyl-H), 1.70–1.60 (m, 6H, hexyl-H), 1.56–1.40 (m, 12H, hexyl-H), 1.16–1.06 (m, 6H, hexyl-H), 0.98 (t, 9 H, ^3J = 7.0 Hz, hexyl-H), −3.65 (br s, 2H, -NH).

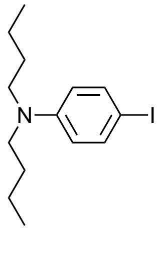

Compound 23: Compound **23** was prepared as per literature procedure (Mohr et al., 1997). 4-Iodoaniline (5.00 g, 22.8 mmol) was mixed with butyl iodide (10 mL, 88.0 mmol) with Na_2CO_3 (8.00 g) in DMF (13 mL). The mixture was degassed and then stirred under Ar for 18 h at 100 °C. The crude mixture was diluted with toluene, washed with water. The crude reaction was again mixed with chloroform and washed with water (3 × 200 mL) and dried over Na_2SO_4. The solvent was evaporated, and the crude material was purified by column chromatography on silica (9:1 40–60 °C petrol ether:CH_2Cl_2). **Yield:** 7.6 g, 100%. **^{1}H NMR** (400 MHz, $CDCl_3$) δ/ppm: 7.41 (d, 2 H, ^3J = 9.09 Hz, Ar-H), 6.41 (d, 2 H, ^3J = 9.17 Hz, Ar-H), 3.22 (t, 4 H, ^3J = 7.53 Hz), 1.53 (m, 4 H), 1.33 (m, 4H), 0.94 (t, 6 H, ^3J = 7.34 Hz). **^{13}C NMR** (100 MHz, $CDCl_3$) δ/ppm: 147.7, 137.7, 114.1, 100.1, 50.8, 29.3, 20.4, 14.1. ***m/z* (ESI+)** 332.0, 333.0 ($C_{14}H_{22}IN$, M+H requires 332.0, M+2H requires 333.0).

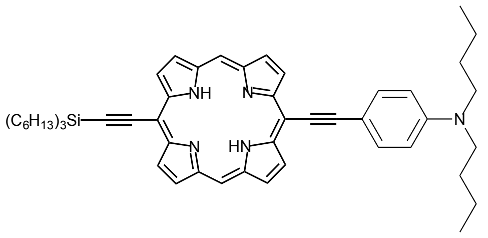

Porphyrin 24: 5-Ethynyl-15-[(trihexylsilyl)ethynyl]porphyrin **22**, (140 mg, 0.218 mmol), $Pd_2(dba)_3$ (22 mg, 0.021 mmol), PPh_3 (25 mg, 0.095 mmol), and CuI (5 mg, 0.026 mmol) were transferred and dried in a Schlenk tube in vacuo for 1 h. DIPA (8 mL) and toluene (8 mL) were added and the reaction mixture thoroughly freeze-pump-thaw degassed (3 cycles). 4-Iodo-*N*,*N*-dibutylaniline **23** (721 mg, 2.18 mmol) was added to the reaction mixture and the mixture was stirred at 50 °C for 1 h under Ar. Progress of the reaction was monitored by TLC (PET ether 40–60 °C : EtOAc 10 : 1). Upon completion, the mixture was passed through a silica plug (CH_2Cl_2), concentrated and purified by flash chromatography on SiO_2 (PET ether 40–60 °C : CH_2Cl_2 20 : 1 : 1 to 10 : 1 : 1 to 5: 1 : 1). Porphyrin **24** was obtained as a green glass. **Yield:** 172 mg, 93%. **^{1}H NMR** (400 MHz, $CDCl_3$) δ/ppm: 9.91 (s, 2H, meso-H), 9.65 (m, 2H, **β**-H), 9.58 (d, 2H, ^3J = 4.3 Hz, **β**-H), 9.18 (m, 4H, **β**-H), 7.92 (d, 2H, ^3J = 8.6 Hz, aniline-H), 6.84 (d, 2H, ^3J = 8.6 Hz, aniline-H), 3.42 (t, 4H, ^3J = 7.8 Hz ,butyl-H), 1.90–1.80 (m, 6H, hexyl-H), 1.77–1.38 (m, 26H, butyl-H, hexyl-H), 1.13–1.03 (m, 12H, butyl-H, hexyl-H), 0.97 (t, 9H, ^3J = 6.8 Hz, hexyl-H), −2.83 (br s, 2H, -NH). ***m/z* (MALDI-ToF)**: 843.57, 844.56, 845.56 ($C_{56}H_{73}N_5Si$, M requires 843.56, M+H requires 844.56, M+2H requires 845.56).

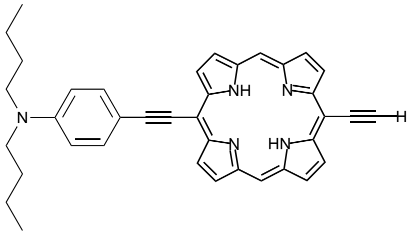

Porphyrin 25: Intermediate porphyrin **25** was prepared as follows: TBAF (1.0 M in THF, 0.402 mL, 0.402 mmol) was added to a solution of **24** (170 mg, 0.201 mmol) in CH_2Cl_2 (30 mL) and stirred for 20 min at RT. The reaction mixture was passed through a silica plug (CH_2Cl_2) and evaporated to dryness to give **25**. **Yield:**112 mg, 100%. The crude product mixture contained trihexylsilane as byproduct. The crude product mixture was taken forward for Sonogashira coupling without any further purification because of high reactivity of the product. **^{1}H NMR** (400 MHz, $CDCl_3$) δ/ppm: 10.01 (s, 2H, meso-H), 9.72 (d, 2H, ^3J = 4.5 Hz, **β**-H), 9.63 (d, 2H, ^3J = 4.5 Hz, **β**-H), 9.25 (m, 4H, **β**-H), 7.91 (d, 2H, ^3J = 8.8 Hz, aniline-H), 6.83 (d, 2H, ^3J = 8.8 Hz, aniline-H), 4.20 (s, 1H, acetylene-H), 3.43 (m, 4H, butyl-H), 1.75–1.65 (m, 4H, butyl-H), 1.52–1.42 (m, 4H, butyl-H), 1.05 (t, 6H, ^3J = 7.4 Hz, butyl-H), −2.61 (br s, 2H, -NH).

Porphyrin 1: *N*,*N*-Dibutyl-4-[(15-ethynylporphyrin-5-yl)ethynyl]aniline **25** (112 mg, 0.201 mmol) was mixed with $Pd_2(dba)_3$ (18 mg, 20.1 µmol), PPh_3 (21 mg, 80.0 µmol), CuI (4 mg, 21.0 µmol) and 4-iodopyridine (400 mg, 2.014 mmol) were dried *in vacuo* for 1 h before DIPA (9 mL) and toluene (9 mL) were added and the mixture freeze-pump-thaw degassed. The mixture stirred at 40°C for 3 h under Ar. Upon completion, the mixture was passed through a silica plug (CH_2Cl_2 with 5% MeOH) then purified by flash chromatography (CH_2Cl_2:THF 5:1 to 3:1) and the fractions were evaporated to dryness. The product mixture was recrystallized (MeOH layered over $CHCl_3$) to give **1** as a green solid. **Yield:** 115 mg, 90%. ^{1}**H NMR** (400 MHz, $CDCl_3$) δ/ppm: 9.89 (s, 2H, meso-H), 9.66 (m, 2H, **β**-H), 9.52 (m, 2H, **β**-H), 9.16 (m, 4H, **β**-H), 8.84 (m, 2H, pyridine-H), 7.91 (d, 2H, ^3J = 8.8 Hz, aniline-H), 7.88 (m, 2H, pyridine-H), 6.84 (d, 2H, ^3J = 8.8 Hz, aniline-H), 3.44 (m, 4H, butyl-H), 1.77–1.67 (m, 4H, butyl-H), 1.53–1.43 (m, 4H, butyl-H), 1.05 (t, 6H, ^3J = 7.4 Hz, butyl-H), −2.61 (br s, 2H, -NH). ***m/z* (MALDI-ToF)**: 638.89, 639.84, 630.79 ($C_{43}H_{38}N_6$, M requires 638.31, M+H requires 639.31, M+2H requires 640.31). **UV-Vis** (DMF, 25 °C) λ_{max} (log **ε**): 692 nm (4.36), 614 nm (4.41), 422 nm (4.89).

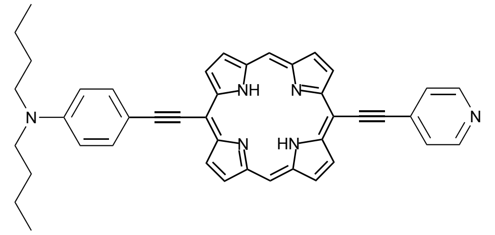

Porphyrin AK-1: Porphyrin **1** (15 mg, 22.5 µmol) was mixed with *N*[1]-(3-iodopropyl)-*N*[1],*N*[1],*N*[3],*N*[3],*N*[3]-pentamethylpropane-1,3-diaminium-di(hexafluorophosphate) **3** (600 mg, 1 mmol, 45 eq.) and dried under high vacuum at 50 °C for 4 h. Dry dimethylacetamide (1.5 mL) was added to the mixture and the reaction mixture was stirred at 115 °C for 6 h in inert atmosphere. TLC (20% THF in DCM) confirmed the consumption of starting material. Solvent was evaporated from crude mixture which was then purified by size-exclusion column chromatography (SX-1 beads in DMF). The second band (product) was passed through a Dowex 1X8 chloride form ion-exchange chromatography column. The reaction mixture was then sequentially washed with water (3 × 30 mL), MeOH (3 × 30 mL) and diethyl ether (1 × 30 mL). The process of ion-exchange and washing was repeated. In the end, the reaction mixture was again passed through the size-exclusion column chromatography (SX-1 beads in DMF). The solvent was evaporated under reduced pressure to yield the product **AK-1** as green solid. **Yield:** 11 mg, 50%. ^{1}**H NMR** (500 MHz, d_6–DMSO at 50 °C) δ/ppm: 10.46 (s, 2H, meso-H), 9.90 (d, 2H, ^3J = 4.5 Hz, β-H), 9.78 (d, 2H, ^3J = 4.5 Hz, β-H), 9.65 (d, 2H, ^3J = 4.5 Hz, β-H), 9.56 (d, 2H, ^3J = 4.5 Hz, β-H), 9.28 (d, 2H, ^3J = 6.1 Hz, pyridine-H), 9.00 (d, 2H, ^3J = 6.1 Hz, pyridine-H), 7.99 (d, 2H, ^3J = 8.4 Hz, aniline -H), 6.92 (d, 2H, ^3J = 8.4 Hz, aniline-H), 4.77 (t, 2H, ^3J = 4.4 Hz, CH_2) 3.52 (m, 2H, CH_2), 3.35 (t, 4H, ^3J = 7.6 Hz, butyl-H), 3.36 (m, 4H, $2CH_2$), 3.15 (m, 15 H, methyl-H), 2.64–2.54 (m, 2H, CH_2), 2.28–2.18 (m, 2H, CH_2), 1.68–1.58 (m, 4H, butyl-H), 1.49–1.39 (m, 4H, butyl-H), 1.01 (t, 6H, ^3J = 7.4 Hz, butyl-H). ***m/z* (MALDI-ToF)**: 1114.96 ($C_{54}H_{65}N_8F_{12}P_2$, M requires 1115.46). **UV-Vis** (DMF, 25 °C) λ_{max} (log **ε**): 709 nm (4.37), 631 nm (4.24), 440 nm (4.67). **Quantum yield** φ_f (DMF, 25 °C): 0.0033.

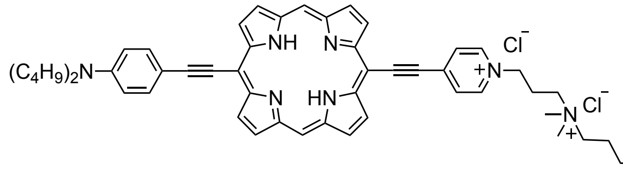

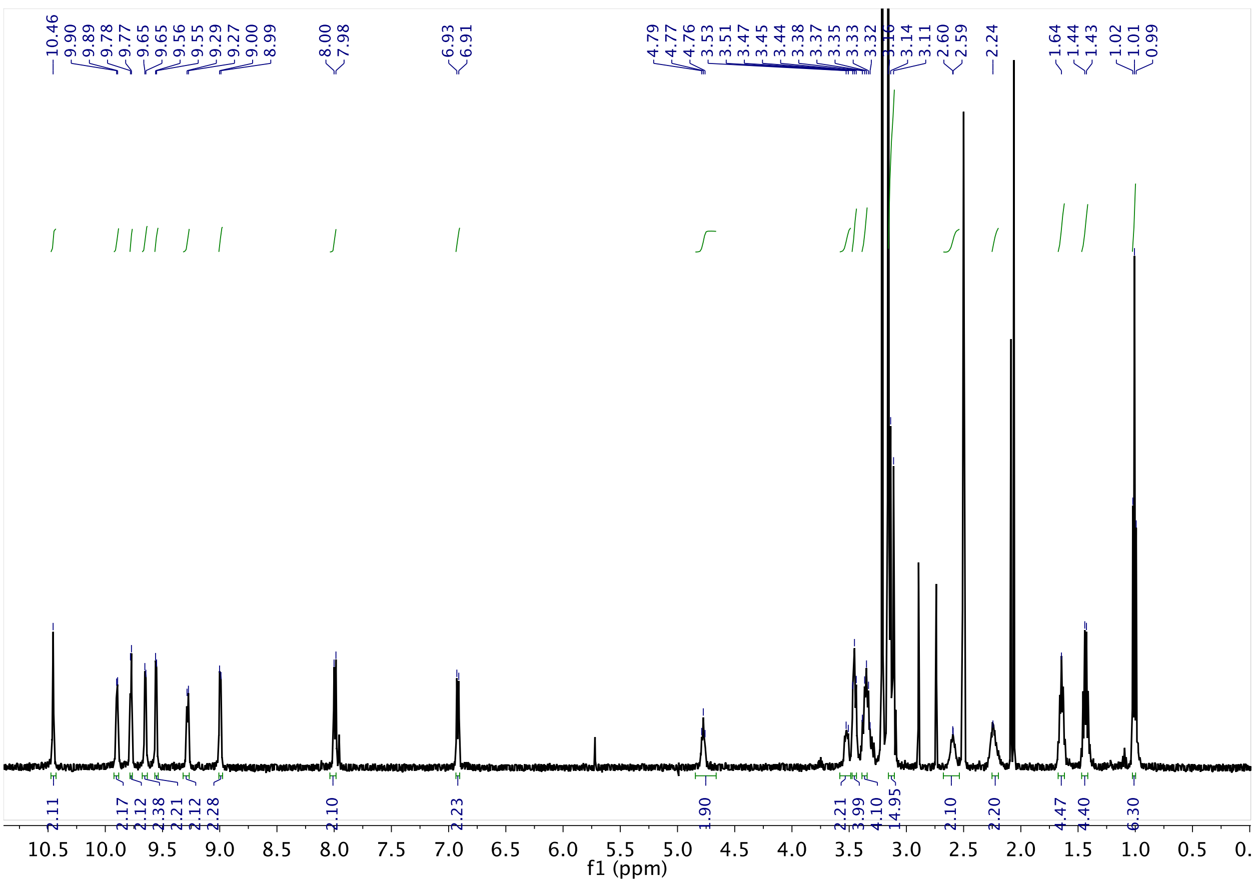

Figure S11. ^{1}H-NMR spectrum of **AK-1** (d_6–DMSO, 50 °C, 500 MHz, DOSY experiment), related to Figure 1.

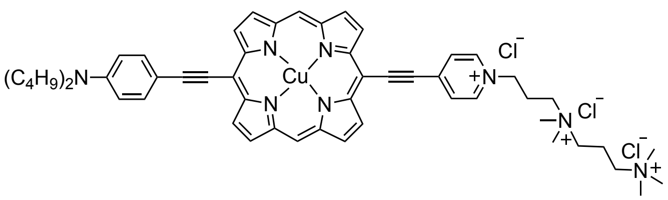

Porphyrin AK-1.Cu: Porphyrin **AK-1** (7 mg, 7.5 µmol) was dissolved in DMF (1 mL). Excess of copper(II) acetate monohydrate (30 mg) was dissolved in MeOH (1 mL) and mixed with the DMF containing porphyrin. The mixture was heated for 8 h at 50 °C after which the solvent was evaporated under reduced pressure. The formation of the product was confirmed by UV-Vis spectroscopy. The crude mixture was re-dissolved in DMF (0.5 mL) and passed through a small plug of SX-1 beads. The solvent was evaporated, and the porphyrin was washed with methanol and distilled water two times each. The purified product was dried under high vacuum overnight. **Yield:** 5.0 mg, 67%. ***m/z* (MALDI-ToF)**: 1176.27 ($C_{54}H_{63}N_8F_{12}P_2Cu$, M requires 1176.37). **UV-Vis** (DMF, 25 °C) λ_{max} (log **ε**): 668 nm (4.51), 441 nm (4.74).

3.3 Synthesis of IG-1

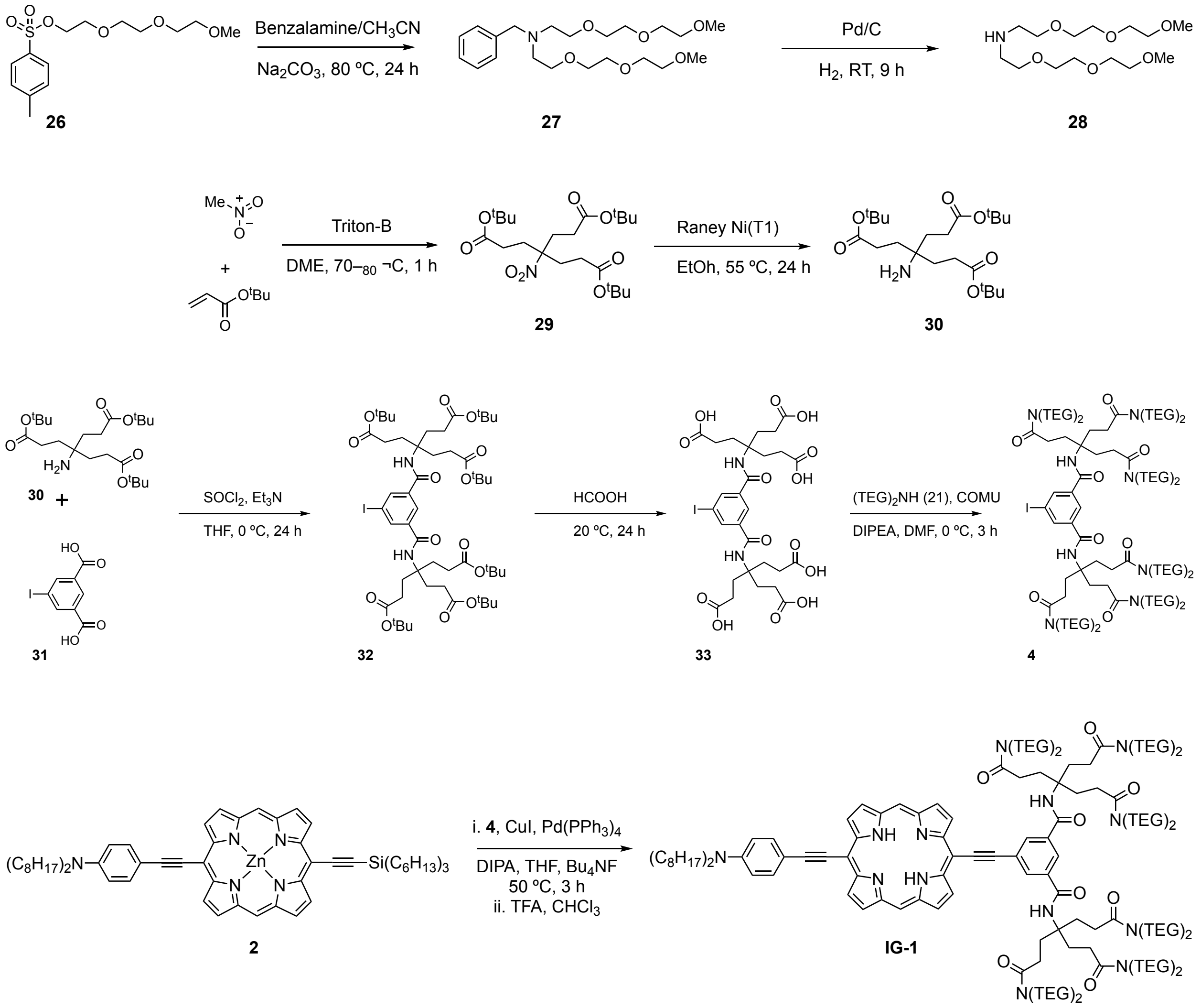

Scheme S3. Synthetic procedure for **IG-1**, related to Figure 1.

Compounds **26**, **27**, **28**, **29**, and **30** were synthesized according to the literature procedures (Dominguez et al., 1961; Newkome et al., 1991; Selve et al., 1991; Snow and Foos, 2003).

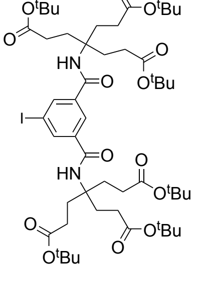

Compound 32: Iodoisophthalic acid **31** (500 mg, 1.7 mmol) was refluxed in $SOCl_2$ (15 mL, 200 mmol) for 16 h. Excess of thionyl chloride was removed by distillation and the resulting acid chloride was dried under high vacuum. The 3-iodoisophthalic acid chloride (563 mg, 1 eq.) was not characterized and instead it was dissolved in dry THF (3 mL) and used immediately in the following peptide coupling step. The solution of Behera's amine **30** (1.56 g, 2.2 eq.) with Et_3N (277 μL) was also prepared in dry THF (3 mL) and added dropwise to the solution of bis-acid chloride. Reaction mixture was left to stir overnight at room temperature. The reaction mixture was concentrated to form a viscous yellow crude oil. Purification was carried out by flash chromatography on SiO_2 (PET ether 40–60 °C:EtOAc 10:1 to 5:1 to neat EtOAc). The fraction containing the product was concentrated and dried under high vacuum overnight to afford **32** as a white

crystalline powder. **Yield:** 702 mg, 37.5%. ^{1}**H NMR** (400 MHz, $CDCl_3$) δ/ppm: 8.30 (d, 2H, J = 1.6 Hz, Ar-H), 8.28 (t, 1H, J = 1.4 Hz, Ar-H), 7.31 (s, 2H, Amide-H), 2.30 (t, 12H, J = 7.4 Hz, CH_2) 2.12 (t, 12H, J = 7.4 Hz, CH_2), 1.44 (s, 54H, *t*-Bu).

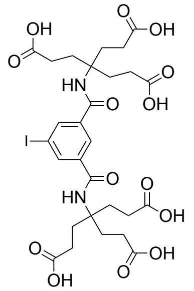

Compound 33: Compound **32** (200 mg, 184 µmol, 1 eq.) was dissolved in 98% formic acid (8.1 mL) and left stirring at room temperature for 24 h. On the next day, the solution was concentrated and toluene (8 mL) was added to help azeotropically remove the residual formic acid. On evaporation, the product **33** was obtained as a white powder. **Yield:** 138 mg. ^{1}**H NMR** (400 MHz, DMSO-d_6) δ/ppm: 12.24 (br. s, 6H, Acid-H), 8.26 (m, 2H, Ar-H), 8.15 (m, 1H, Ar-H), 7.75 (br. s, 2H, Amide-H), 2.17 (t, 12H, J = 7.6 Hz, CH_2), 1.98 (t, 12H, J = 8.8 Hz, CH_2).

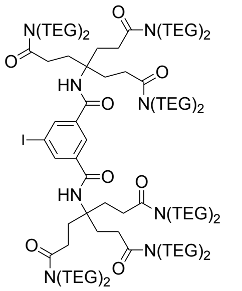

Compound 4: 3-Iodoisophthalic acid **33** (50 mg, 67 µmol, 1 eq.) was dissolved in dry DMF (0.2 mL) and cooled in an ice bath to 0 °C. In parallel compound **28** (247 mg, 800 µmol, 12 eq.) was dissolved in dry DMF (0.2 mL) and also cooled to 0 °C. To each cooled solution DIPEA (0.070 mL, 800 µmol, 12 eq.) was added. Next, to the solution of compound **33**, COMU (El-Faham and Albericio, 2010) coupling reagent (218 mg, 800 µmol, 12 eq.) was added and stirred for 1 min before the solution of amine was added dropwise. Combined solutions were stirred for 1 h at 0 °C and an additional 2 h at room temperature. Crude reaction mixture was worked up by diluting with EtOAc (20 mL) and a following washing with HCl (1.0 M, 2 × 5 mL), $NaHCO_3$ (1.0 M, 2 × 5 mL) and saturated NaCl (2 × 5 mL). The aqueous phase was additionally washed with DCM (4 × 200 mL) (until no more UV active compound partitioned into DCM) which was then combined with the organic phase. The product was purified by size-exclusion chromatography ($CHCl_3$) to obtain **4** as an oil. **Yield:** 131 mg, 78 %. ^{1}**H NMR** (400 MHz, $CDCl_3$) δ/ppm: 8.76 (br s, 2H, *amide*-NH), 8.40 (s, 1H, Ar-H), 8.30 (s, 2H, Ar-H), 3.63–3.47 (br m, 74H, TEG-CH_2), 3.35 (s, 9H, TEG-OCH_3), 3.33 (s, 9H, TEG-OCH_3), 2.46 (t, 6H, J = 6.2 Hz, CH_2), 2.15 (t, 6H, J = 6.2 Hz, CH_2).

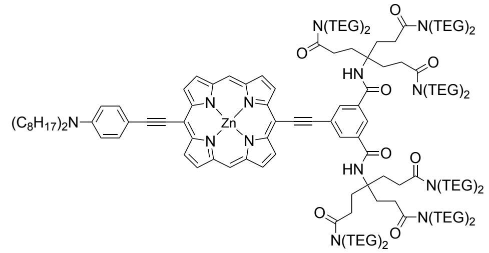

Porphyrin IG-1.Zn: Trihexylsilylacetylene,15-ethynyl porphyrin **2** (21 mg, 20.6 µmol, 1.5 eq.) and $Pd(PPh_3)_4$ (4.16 mg, 3.6 µmol, 0.2 eq.), CuI (0.7 mg, 3.6 µmol, 0.2 eq.) and compound **4** (30 mg, 12 µmol, 1 eq.) were transferred and dried in a Schlenk tube *in vacuo* for 1 h. THF (0.5 mL) and DIPA (0.5 mL) was added and the reaction mixture thoroughly freeze-pump-thaw degassed (4 cycles). Bu_4NF (0.18 mL, 180 µmol, 1 M in THF, 15 eq.) was added to the reaction mixture and the mixture was freeze-pump-thaw degassed again (another 2 cycles) then brought to 50 °C and stirred for 3 h under N_2. Progress of the reaction was monitored by TLC (PET ether 40–60 °C:EtOAc:Py 10:1:1). On completion the mixture was passed through a silica plug (PET ether 40–60 °C:EtOAc 3:1), concentrated and purified by flash chromatography on SiO_2 (PET ether 40–60 °C: CH_2Cl_2:Py 20:1:1 to 10:1:1 to pure CH_2Cl_2). Product **IG-1.Zn** was obtained as a green solid. **Yield**: 30 mg, 80%. ^{1}**H NMR** (400 MHz, $CDCl_3$) δ/ppm: 9.97 (s, 2H, *meso*-CH), 9.78 (m, 4H, β-CH), 9.24 (m, 4H, β-CH), 8.80 (br s, 2H, *amide*-NH), 8.58 (s, 2H, *Ar*-H), 8.53 (s, 1H, *Ar*-H), 7.84 (d, 2H, J = 8.8 Hz, $Ar_{aniline}$-H), 6.75 (d, 2H, J = 8.9 Hz, $Ar_{aniline}$-H), 3.61–3.23 (s, 148H, *TEG(CH₃)*-H), 3.20 (s, 18H, *TEG(CH₃)*-H), 3.18 (s, 18H, *TEG(CH₃)*-H), 2.50 (t, 12H, J = 6.8 Hz, CH_2), 2.22 (t, 12H, J = 6.4 Hz, CH_2), 1.63 (m, 6H, *octyl*-CH_2), 1.38–1.21 (m, 22H, *octyl*-CH_2), 0.85 (t, 6H, *octyl*-CH_3). ***m/z* (MALDI-TOF):** 3129.85 ($[M+Na]^+$ 100%, $C_{158}H_{257}N_{13}O_{44}ZnNa^+$ requires 3129.75).

Porphyrin IG-1: Compound **IG-1.Zn** (5.0 mg, 1.6 μmol, 1 eq.) was dissolved in $CHCl_3$ (0.5 mg) in a dry round bottom flask. TFA (12.5 μL, 100 eq.) was added at once to the solution of the porphyrin. Reaction was allowed to proceed for 15 min. On completion, the reaction was stopped by pouring the reaction mixture into a flask with large volume of $CHCl_3$ (50 mL) and washing the resultant diluted solution with saturated solution of $NaHCO_3$ until basic pH is reached. Organic phase was separated and dried with $MgSO_4$, then filtered and concentrated. Product was obtained as a dark green solid. **Yield:** 3.5 mg, 70 %. **^{1}H NMR** (400 MHz, $CDCl_3$) δ/ppm: 10.05 (s, 2H, *meso*-CH), 9.71 (m, 4H, β-CH), 9.27 (d, 2H, J = 4.6 Hz, β-CH), 9.24 (d, 2H, J = 4.5 Hz, β-CH), 8.86 (br s, 2H, *amide*-NH), 8.61 (s, 2H, *Ar*-H), 8.58 (s, 1H, *Ar*-H), 7.84 (d, 2H, J = 8.4 Hz, $Ar_{aniline}$-H), 6.75 (d, 2H, J = 8.9 Hz, $Ar_{aniline}$-H), 3.6–3.23 (m, 160 H, *TEG(CH₂-CH₃)*), 2.51 (t, 12H, J = 6.6 Hz, CH_2), 2.22 (t, 12H, J = 6.5 Hz, CH_2), 1.64 (m, 6H, *octyl*-CH_2), 1.38–1.21 (m, 22H, *octyl*-CH_2), 0.86 (t, 6H, *octyl*-CH_3), –2.28 (s, 4H, NH-*ring*). ***m/z* (MALDI-TOF):** 3066.78 ([$C_{158}H_{259}N_{13}O_{44}Na^+$ $[M+Na]^+$ requires 3066.84). **UV-Vis** (DMF, 25 °C) λ_{max} (log **ε**): 430 nm (4.83); 614 nm (4.34); 692 nm (4.30).

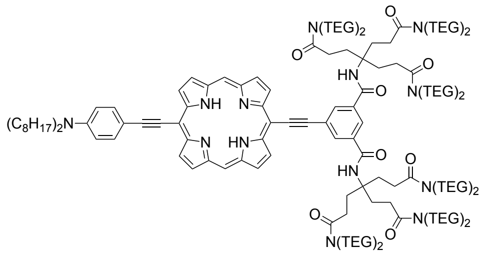

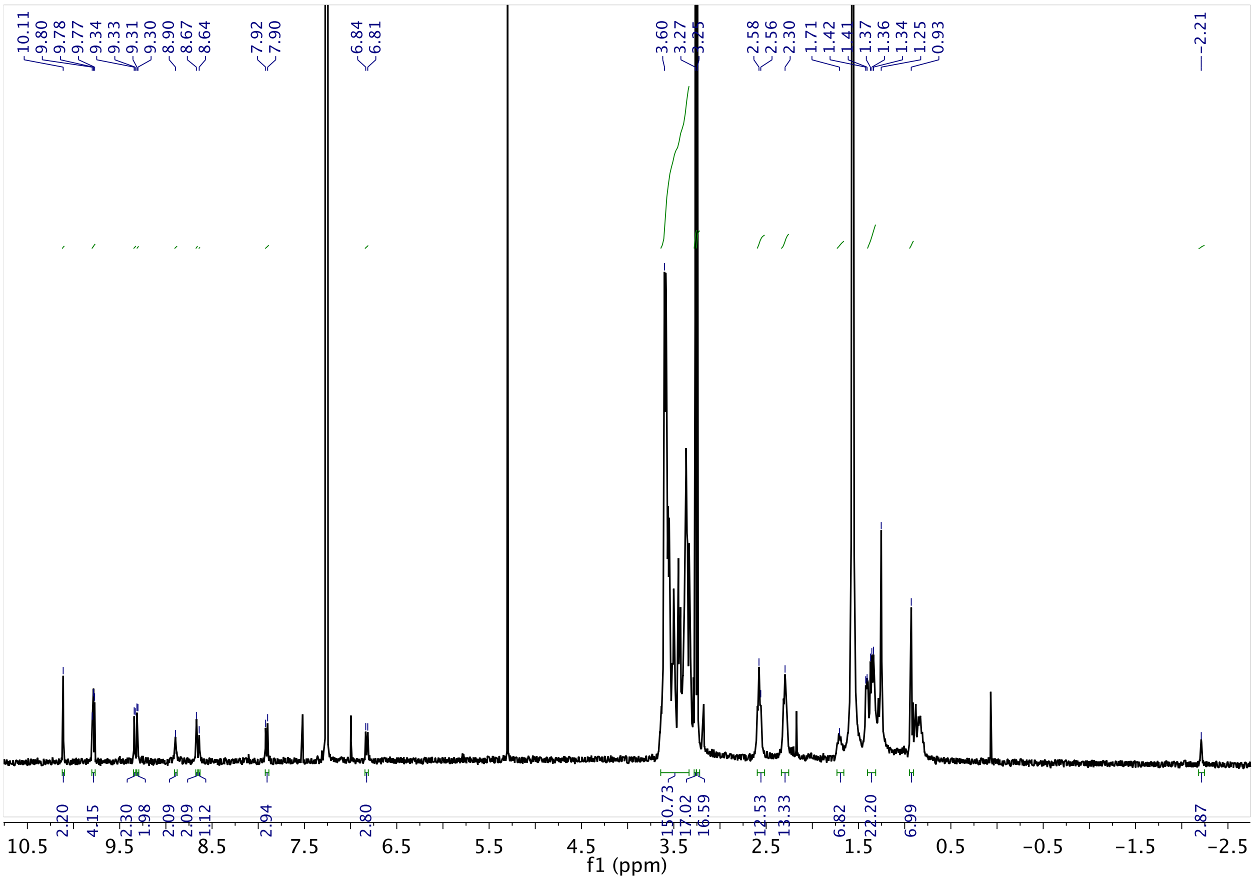

Figure S12: ^{1}H-NMR spectrum of **IG-1** ($CDCl_3$, 400 MHz), related to Figure 1.

3.4 Synthesis of JW-1

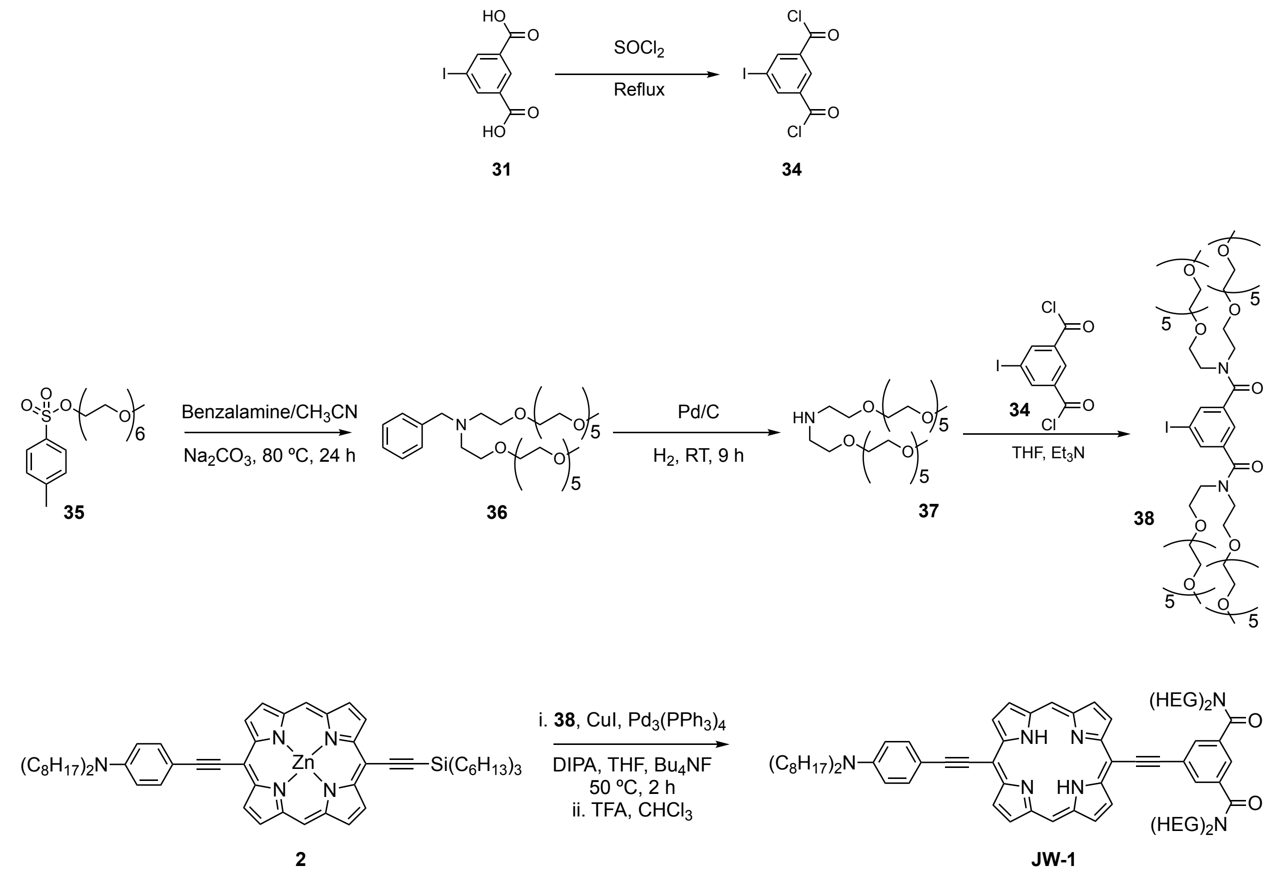

Scheme 4. Synthetic procedure for **JW-1**, related to Figure 1.

Compounds **35**, **36**, and **37** were synthesized as per the protocol followed during synthesizing intermediates for **IG-1**.

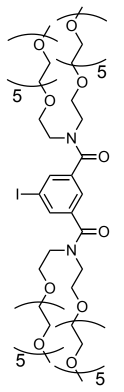

Compound 38: Iodoisophthalic acid **31** (0.50 g, 1.7 mmol) was refluxed in $SOCl_2$ (15.0 mL, 207 mmol) for 16 h to form **34**. $SOCl_2$ was removed under reduced pressure and the brown/red oily residue was dried under high vacuum for several hours. The oil was stored under N_2 and used within 24 h. The oil (81 mg, 0.25 mmol) was dissolved in THF (1.7 mL) and added dropwise to a solution of **37** (340 mg, 0.59 mmol) in THF (1.7 mL) and Et_3N (83 µL, 0.59 mmol) at 0 °C. The reaction was stirred at 20 °C for 3 h and then the precipitate was filtered, and the solvent was evaporated under reduced pressure. The crude residue was dissolved in CH_2Cl_2, washed with 1.0 M aq. HCl and extracted with CH_2Cl_2. The organic layers were dried over $MgSO_4$ and filtered. Size-exclusion chromatography used to purify the product to yield **38**. **Yield**: 0.26 g, 73 %. **^{1}H NMR** (400 MHz, d_6–DMSO) δ/ppm: 7.74 (d, J = 1.2 Hz, 2 H), 7.31 (t, J = 1.2 Hz, 1 H), 3.36–3.72 (m, 96 H), 3.31 (s, 12 H). **^{13}C NMR** (100 MHz, d_6–DMSO) δ/ppm: 169.7, 138.8, 136.5, 124.7, 93.6, 71.9, 70.6, 70.5, 70.4, 69.1, 68.6, 59.0, 49.8, 45.0. ***m/z* (ESI+)** 724.3110 ($C_{60}H_{111}IN_2O_{26}$ $(M + 2Na)^{2+}$: 724.3127 requires 724.3110).

Porphyrin JW-1.Zn: To a pre-dried Schlenk tube were added porphyrin **2** (25 mg, 25 µmol), **38** (35 mg, 25 µmol), $Pd(PPh_3)_4$ (2.9 mg, 2.5 µmol) and CuI (0.5 mg, 3 µmol). These were dried under vacuum for 30 mins, then the flask purged with N_2 to allow addition of THF (1 mL) and DIPA (1 mL). The mixture was freeze pump-thaw degassed 3 times, then Bu_4NF (1.0 M solution in THF, 0.25 mL, 0.25 mmol) was added and the reaction heated to 50 °C under N_2. After 2 h, the reaction was passed through a column of silica, eluting with THF : 1% pyridine then $CHCl_3$: 10% MeOH : 1% pyridine. The crude mixture was concentrated and purified by size-exclusion chromatography ($CHCl_3$) to isolate the desired product as a green solid after drying. **Yield:** 25 mg, 50%. **^{1}H NMR** (400 MHz, $CDCl_3$) δ/ppm: 10.05 (s, 2 H, meso-H), 9.85 (d, J = 4.3 Hz, 2 H, β-H), 9.78 (d, J = 4.5 Hz, 2 H, β-H), 9.33 (d, J = 4.5 Hz, 2 H, β-H), 9.31 (d, J = 4.3 Hz, 2 H, β-H), 8.14 (d, J = 1.5 Hz, 2 H, Ar-*ortho*-H), 7.91 (d, J = 8.8 Hz, 2 H, aniline-H), 7.52 (t, J = 1.5 Hz, 1 H, Ar-*para*-H), 6.82 (d, J = 9.1 Hz, 2 H, aniline), 3.10–3.93 (m, 112 H, HEG, N-CH_2-C_7H_{15}, O-CH_3), 1.29–1.44 (m, 20 H), 0.93 (t, J = 6.3 Hz, 6 H). **^{13}C NMR** (125 MHz, $CDCl_3$ with 1% d_5-pyridine) δ/ppm: 170.9, 152.1, 151.8, 149.2, 148.2, 137.7, 133.0, 132.4, 131.8, 131.4, 130.8, 130.5, 125.0, 124.7, 111.5, 109.6, 107.7, 102.8, 98.4, 97.6, 94.9, 94.2, 91.0, 71.8, 71.8, 70.6, 70.5, 70.3, 70.2, 70.2, 69.2, 69.0, 59.0, 58.9, 51.1, 49.9, 45.3, 31.8, 29.5, 29.3, 27.3, 27.2, 22.7, 14.1. MS Calcd for. ***m/z* (MALDI-TOF):** 2034.94 ($C_{106}H_{159}N_7O_{26}Zn$ [M + Na] requires 2035.05).

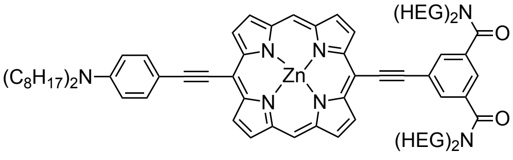

Porphyrin JW-1: Porphyrin **JW-1.Zn** (10 mg, 4.9 µmol) was dissolved in $CHCl_3$ (4.4 mL) and the solution was stirred. TFA (88 mL, 1.2 mmol) was added and the reaction stirred for further 1 h, after which aq. sat. $NaHCO_3$ was added (2 mL). The product was washed with water (2 × 5 mL), extracted with $CHCl_3$ (2 × 5 mL), dried over $MgSO_4$ and concentrated. The product was precipitated as a film by addition of 60–80 petrol ether to a CH_2Cl_2 solution, followed by careful evaporation of the CH_2Cl_2 and addition of pentane, yielding the clean product **JW-1**. **Yield:** 8.7 mg, 90%. **^{1}H NMR** (400 MHz, $CDCl_3$) δ/ppm: 10.09 (s, 2 H, meso-H), 9.75 (d, J = 4.4 Hz, 2 H, β-H), 9.69 (d, J = 4.7 Hz, 2 H, β-H), 9.32 (d, J = 4.6 Hz, 2 H, β-H), 9.29 (d, J = 4.3 Hz, 2 H, β-H), 8.14 (br s, 2 H, Ar-*ortho*-H), 7.91 (d, J = 8.5 Hz, 2 H, aniline-H), 7.56 (s, 1 H, Ar-*para*-H), 6.82 (d, J = 8.9 Hz, 2 H, aniline-H), 3.26–3.93 (m, 112 H, HEG, N-CH_2-C_7H_{15}, O-CH_3), 1.68–1.78 (m, 9 H), 1.23–1.42 (m, 33 H), 0.93 (t, J = 6.8 Hz, 6 H), –2.27 (br. s., 2 H, N-H). **^{13}C NMR** (125 MHz, $CDCl_3$) δ/ppm: 169.8, 147.5, 144.1, 136.7, 132.3, 131.3, 130.6, 129.8, 129.4, 128.8, 124.2, 123.4, 110.5, 107.9, 106.0, 102.1, 99.4, 96.9, 94.4, 91.7, 88.5, 70.9, 70.8, 70.8, 69.6, 69.5, 69.5, 69.3, 69.3, 69.2, 68.2, 67.9, 58.0, 57.9, 52.4, 50.1, 48.9, 44.3, 30.8, 28.5, 28.3, 26.3, 26.2, 21.7, 13.1. ***m/z* (MALDI-TOF):** 1971.77 ($C_{106}H_{161}N_7O_{26}Na$, (M + Na) requires1972.14). **UV-Vis** (DMF, 25 °C) λ_{max} (log **ε**): 425 nm (5.01); 615 nm (4.57); 693 nm (4.53).

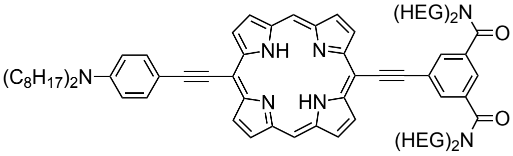

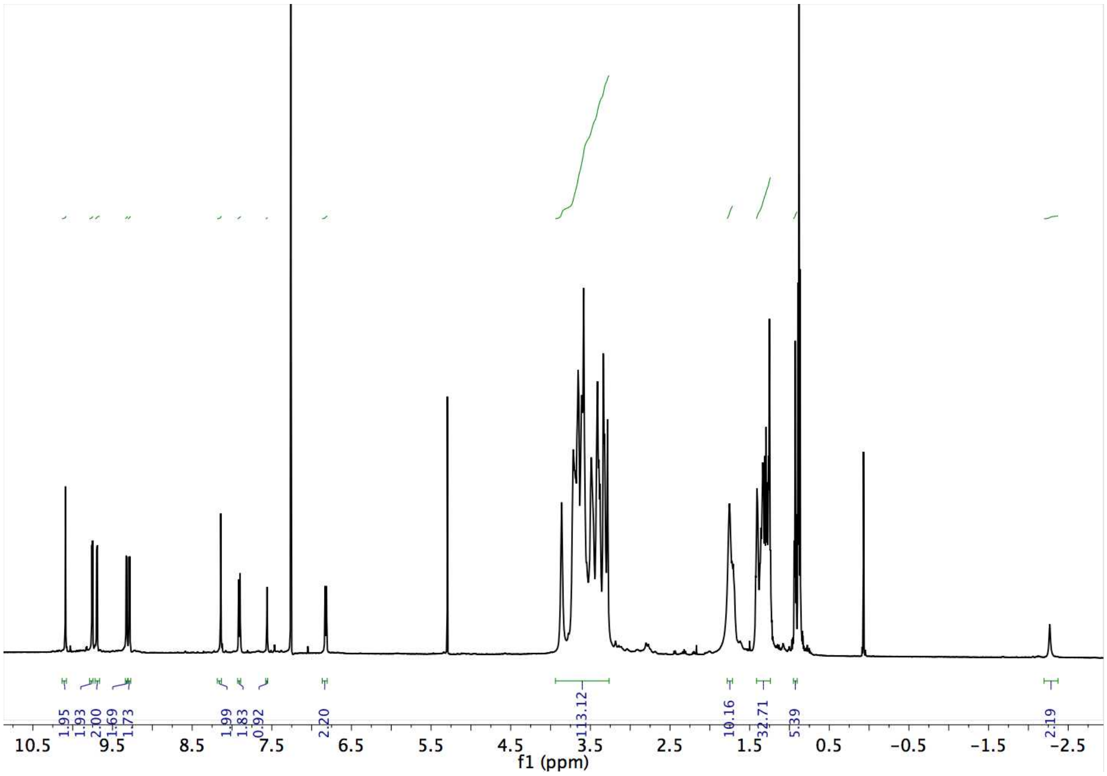

Figure S13. ^{1}H-NMR spectrum of **JW-1** ($CDCl_3$, 400 MHz), related to Figure 1.

Supplemental References

Anderson, H.L., 1992. Meso-alkynyl porphyrins. Tetrahedron Lett. 33, 1101–1104.

Balaz, M., Collins, H.A., Dahlstedt, E., Anderson, H.L., 2009. Synthesis of hydrophilic conjugated porphyrin dimers for one-photon and two-photon photodynamic therapy at NIR wavelengths. Org. Biomol. Chem. 7, 874–888.

Dominguez, X.A., Lopez, I.C., Franco, R., 1961. Notes: Simple Preparation of a Very Active Raney Nickel Catalyst. J. Org. Chem. 26, 1625–1625.

El-Faham, A., Albericio, F., 2010. COMU: A third generation of uronium-type coupling reagents. J. Pept. Sci. 16, 6–9.

Ka, J.-W., Lee, C.-H., 2000. Optimizing the synthesis of 5,10-disubstituted tripyrromethanes. Tetrahedron Lett. 41, 4609–4613.

Littler, B.J., Miller, M.A., Hung, C.H., Wagner, R.W., O'Shea, D.F., Boyle, P.D., Lindsey, J.S., 1999. Refined synthesis of 5-substituted dipyrromethanes. J. Org. Chem. 64, 1391–1396.

Lopez-Duarte, I., Reeve, J.E., Perez-Moreno, J., Boczarow, I., Depotter, G., Fleischhauer, J., Clays, K., Anderson, H.L., 2013. "Push-no-pull" porphyrins for second harmonic generation imaging. Chem. Sci. 4, 2024–2027.

Mohr, G.J., Lehmann, F., Grummt, U.W., Spichiger-Keller, U.E., 1997. Fluorescent ligands for optical sensing of alcohols: Synthesis and characterisation of p-N,N-dialkylamino-trifluoroacetylstilbenes. Anal. Chim. Acta 344, 215–225.

Newkome, G.R., Moorefield, C.N., Baker, G.R., Behera, R.K., 1991. Cascade Polymers: Syntheses and Characterization of One-Directional Arborols Based on Adamantane. J. Org. Chem. 56, 7162–7167.

Reeve, J.E., Collins, H.A., De Mey, K., Kohl, M.M., Thorley, K.J., Paulsen, O., Clays, K., Anderson, H.L., 2009. Amphiphilic porphyrins for second harmonic generation imaging. J. Am. Chem. Soc. 131, 2758–2759.

Sasaki, S., Yoshizato, M., Kunieda, M., Tamiaki, H., 2010. Cooperative C3- and C13-Substituent Effects on Synthetic Chlorophyll Derivatives. European J. Org. Chem. 2010, 5287–5291.

Sebastiano, R., Gelfi, C., Giorgio Righetti, P., Citterio, A., 2001. Omega-Iodoalkylammonium salts as permanent capillary silica wall modifiers: Comparative analysis of their structural parameters and substituent effects. J. Chromatogr. A 924, 71–81.

Selve, C., Ravey, J.C., Stebe, M.J., El Moudjahid, C., Moumni, E.M., Delpuech, J.J., 1991. Monodisperse perfluoro-polyethoxylated amphiphilic compounds with two-chain polar head - preparation and properties. Tetrahedron 47, 411–428.

Snow, A.W., Foos, E.E., 2003. Conversion of Alcohols to Thiols via Tosylate Intermediates. Synthesis (Stuttg). 2003, 509–512.

Tykwinski, R.R., Schreiber, M., Carlón, R.P., Diederich, F., Gramlich, V., 1996. Donor/Acceptor-Substituted Tetraethynylethenes: Systematic Assembly of Molecules for Use as Advanced Materials. Helv. Chim. Acta 79, 2249–2281.

Yi, S., Leon, W., Vezenov, D., Regen, S.L., 2016. Tightening Polyelectrolyte Multilayers with Oligo Pendant Ions. ACS Macro Lett. 5, 915–918.
